# Implementing facility-based kangaroo mother care services: lessons from a multi-country study in Africa

**DOI:** 10.1186/1472-6963-14-293

**Published:** 2014-07-08

**Authors:** Anne-Marie Bergh, Kate Kerber, Stella Abwao, Joseph de-Graft Johnson, Patrick Aliganyira, Karen Davy, Nathalie Gamache, Modibo Kante, Reuben Ligowe, Richard Luhanga, Béata Mukarugwiro, Fidèle Ngabo, Barbara Rawlins, Felix Sayinzoga, Naamala Hanifah Sengendo, Mariam Sylla, Rachel Taylor, Elise van Rooyen, Jeremie Zoungrana

**Affiliations:** 1 MRC Unit for Maternal and Infant Health Care Strategies, University of Pretoria, Private Bag X323, Arcadia 0007, South Africa; 2 Save the Children, Cape Town, South Africa; 3 Save the Children, Washington, DC, USA; 4 Maternal and Child Health Integrated Program (MCHIP), Washington, DC, USA; 5 Save the Children International, Kampala, Uganda; 6 Save the Children International, Bamako, Mali; 7 Save the Children International, Lilongwe, Malawi; 8 Maternal and Child Health Integrated Program (MCHIP), Kigali, Rwanda; 9 Jhpiego, Washington, DC, USA; 10 Ministry of Health, Kigali, Rwanda; 11 Department of Paediatrics, Gabriel Toure Teaching Hospital, Bamako, Mali

**Keywords:** Delivery of health care, Implementation, Infant premature, Kangaroo mother care, Neonatal mortality, Newborn health

## Abstract

**Background:**

Some countries have undertaken programs that included scaling up kangaroo mother care. The aim of this study was to systematically evaluate the implementation status of facility-based kangaroo mother care services in four African countries: Malawi, Mali, Rwanda and Uganda.

**Methods:**

A cross-sectional, mixed-method research design was used. Stakeholders provided background information at national meetings and in individual interviews. Facilities were assessed by means of a standardized tool previously applied in other settings, employing semi-structured key-informant interviews and observations in 39 health care facilities in the four countries. Each facility received a score out of a total of 30 according to six stages of implementation progress.

**Results:**

Across the four countries 95 per cent of health facilities assessed demonstrated some evidence of kangaroo mother care practice. Institutions that fared better had a longer history of kangaroo mother care implementation or had been developed as centres of excellence or had strong leaders championing the implementation process. Variation existed in the quality of implementation between facilities and across countries. Important factors identified in implementation are: training and orientation; supportive supervision; integrating kangaroo mother care into quality improvement; continuity of care; high-level buy in and support for kangaroo mother care implementation; and client-oriented care.

**Conclusion:**

The integration of kangaroo mother care into routine newborn care services should be part of all maternal and newborn care initiatives and packages. Engaging ministries of health and other implementing partners from the outset may promote buy in and assist with the mobilization of resources for scaling up kangaroo mother care services. Mechanisms for monitoring these services should be integrated into existing health management information systems.

## Background

Preterm birth, or birth before 37 completed weeks gestation, is globally the direct leading cause of the estimated three million neonatal deaths each year and the second leading cause of all deaths in children under the age of five [1,2]. Preterm infants’ higher risk of bacterial sepsis adds to the risk of dying [3]. Many of these deaths are preventable through accessible interventions. Kangaroo mother care (KMC) is one such intervention that can substantially contribute to decreasing the risk of death in neonates weighing less than 2000 g [4,5]. Compared with incubator care, KMC has been found to reduce infection (including sepsis), hypothermia, severe illness and lower respiratory tract disease in infants at discharge or 40–41 weeks’ postmenstrual age and at latest follow up. There is also an association with an increase in some measures of infant growth (weight, head circumference, weight gain), breastfeeding, and mother-infant attachment [5].

KMC is a complex intervention that involves three main components present in a supportive environment: placing the infant skin-to-skin against the mother’s chest; exclusive breastfeeding where possible; and early discharge from the health facility, with appropriate follow-up systems [6]. The main modes of KMC practice are continuous KMC (24 hours per day) and intermittent KMC (recurrent but not continuous skin-to-skin contact between mother and baby, a few times a day) over a variable number of days [7].

In recent years many ministries of health have collaborated with development partners and health professionals to systematically introduce, strengthen, or promote the scale up of facility-based KMC. Factors that received attention include: setting KMC policy and service guidelines; developing clinical training materials, supervision schedules and tools; integrating recordkeeping and reporting on KMC into routine monitoring and evaluation systems; documenting implementation; and costing KMC services [7-10]. However, countries had different perspectives when setting and executing their agenda, resulting in varied KMC implementation progress across countries. Approaches to improve quality of care of preterm infants in health facilities was ranked second out of 82 questions in global research priority setting for preterm and low birth weight (LBW) babies [11], demonstrating the importance of understanding sustainable implementation of KMC at scale.

The aim of this study was to provide a systematic ‘snapshot’ of the implementation status of facility-based KMC services in four countries in sub-Saharan Africa to inform further roll-out of KMC and other health system interventions. Other countries and institutions may learn from the barriers and facilitators of KMC institutionalization in these countries.

## Methods

A cross-sectional, mixed-method research design was used to analyse country KMC program performance. Qualitative and quantitative data collection methods were employed, including stakeholder meetings, semi-structured key-informant interviews and observations. Country visits took place between February and May 2012. The research protocol was approved by the Institutional Review Board of the Johns Hopkins Bloomberg School of Public Health (IRB no. 0004134). The ministries of health and all facility directors gave written permission for the study and key informants in facilities gave oral consent before being interviewed and showing the assessors the maternity or neonatal unit where KMC services were being provided.

### Sampling

Sampling for this study took place at two levels. First, four countries – Malawi, Mali, Rwanda, and Uganda – were purposively selected as countries from which potentially rich information could be obtained, on the basis of their perceived progress with KMC implementation and existing platforms for delivering newborn care. Selected newborn statistics for the four countries are presented in Table 1 to give a sense of the burden of preterm birth and to demonstrate differences between countries. All four countries had received financial and/or technical support from the Saving Newborn Lives program and/or the Maternal and Child Health Integrated Program (MCHIP, and its predecessor ACCESS) for the implementation of KMC on its own or as part of essential newborn and/or obstetric care initiatives. In all countries different forms of support were also provided by other agencies or non-governmental organizations at different levels of the health system, either as part of an intervention or on an ad hoc basis.

**Table 1:** Selected national newborn statistics of the four study countries

**Indicator**	**Malawi**	**Mali**	**Rwanda**	**Uganda**
Neonatal mortality rate per 1000 live births^a^[2]	27	49	21	28
Annual number of neonatal deaths^a^[1]	18,000	39,000	9,000	43,000
Preterm birth rate^b^[12]	18%	12%	10%	14%
Neonatal deaths due to preterm complications^b^[1]	36%	33%	34%	38%
Births in a health facility^c^[13]	73%	55%	69%	57%

^a^2011 ^b^2010 ^c^2010–12.

Second, in each country a sample of two groups of informants was selected – stakeholders representing higher-level structures in each country and health facilities representing the grassroots level of KMC implementation. Stakeholders included government, program developers and coordinators, regulatory bodies, professional associations, training and research institutions, health facilities, United Nations and other funding agencies, and non-governmental organizations involved in the improvement of newborn care or the implementation of KMC.

A convenience sample of facilities to receive on-site visits was identified (total n = 39). Logistic and cost constraints did not allow for the use of probability sampling. It was, however, important to ensure that the assessed facilities included all levels of care, where applicable, and also to allow for sufficient geographic spread. Table 2 gives a summary of the types of facilities visited per country. Across all countries combined, more than one in every five facilities reportedly providing KMC services received a site visit, ranging from 100% of facilities in Mali with only seven facilities providing KMC services to 12% in Malawi, where 121 facilities were reported to provide these services.

**Table 2:** Summary of facility samples per country

**Facility information**	**Malawi**	**Mali**	**Rwanda**	**Uganda**	**Total**
Number of facilities reported to provide KMC services, 2011	121	7	30	19	**177**
Facility levels included in sample:					
Central (teaching) hospital	1	1		1	**3**
Regional hospital		3		1	**4**
District hospital	9	3	7	4	**23**
Mission or not-for-profit hospital	1			3	**4**
Rural hospital	1				**1**
(Community) Health centre	2			2	**4**
Number of facilities sampled for the study	14	7	7	11	**39**
Percentage of facilities visited compared to total number reported to provide KMC services	12%	100%	23%	58%	**22%**

### Data collection

In three countries stakeholders were invited to a national plenary meeting to qualitatively solicit their views on KMC implementation. In Rwanda four individual qualitative interviews with stakeholder members served the same purpose. The number of meeting delegates ranged between 11 and 13 per country. Meetings were facilitated by a local representative of Save the Children and the agenda included feedback on the history and status of KMC implementation in the country and a discussion on the role of partners in KMC implementation, strengths and challenges, and recommendations for the way forward. The notes from these meetings and interviews were meant to complement the facility assessments by providing the back-drop and broader framework within which to understand the findings from the facility assessments.

Facilities’ KMC services were assessed by means of a standardized, key-informant interview questionnaire and observation inventory [14] with quantitative and qualitative components that cover the following aspects of service and types of practices: the health care facility (including its baby-friendly status); neonatal and KMC facilities; skin-to-skin practices; history of KMC implementation; involvement of internal role players; physical and financial resources; KMC space (continuous and intermittent KMC); feeding and weight monitoring; referral, discharge and follow up; record keeping and documentation (including availability of job aids, counselling materials, and other tools); KMC education; staffing issues (orientation and training; rotations); and strengths and challenges. The tool, available as an online supplement (Additional file 1 ), had previously been applied in South African KMC outreach trials [15,16] and had been adapted for use in Malawi [17], Ghana [18], Nigeria [19], and Indonesia [20].

In each country a team of local assessors with clinical and/or training experience in KMC were trained in the use of the facility tool. Each team consisted of two to three assessors who prepared and visited facilities together. The facility leadership and staff were informed prior to the team’s visit and were requested to prepare a presentation as part of the assessment activities. The assessor team conducted interviews with KMC focal persons and other staff and members of the management team and observed KMC practices and service provision. Before the team left a site they provided verbal feedback to facility representatives and left a written report behind.

All data collection activities were conducted in either English or French, according to the official language of the country or according to the preference of individual informants. In all four countries the local assessors played the role of interpreter during facility visits where informants felt more comfortable to provide information in a local language.

### Data analysis

During the facility visits each assessor completed his or her own questionnaire and any discrepancies between the assessors were resolved through discussion and consensus, with the final analysis consolidated by the lead investigator (A-MB). Descriptive statistics were generated from some of the questionnaire items and an implementation-progress score out of a possible total score of 30 was calculated for each facility. The scoring is divided according to six stages of change, with each stage having a weighted score (see Table 3): creating awareness (2 points); adopting the concept (2 points); taking ownership (mobilizing resources) (6 points); evidence of practice (7 points); evidence of routine and integration (7 points); sustainable practice (6 points) [14].

**Table 3:** **Scoring of facilities**[16]

**Stages and phases**		**Points per stage**	**Cumulative points**
**Pre-implementation phase**			
Stage 1	Creating awareness	2	2
Stage 2	Adopting the concept	2	4
**Implementation phase**			
Stage 3	Taking ownership	6	10
Stage 4	Evidence of practice	7	17
**Institutionalisation phase**			
Stage 5	Evidence of routine and integration	7	24
Stage 6	Sustainable practice	6	30
**TOTAL**		**30 points**	

The results from the analysis of the facility questionnaires and the themes derived from the open-ended items on the questionnaires were then compared with the themes recorded in the stakeholder meetings and interviews in order to get a sense of the development of KMC implementation in each country and of what appeared to be important facilitators and barriers to KMC implementation in each country and across countries. The final interpretation was confirmed by key role players in each country.

## Results

The history of KMC implementation followed different trajectories in the four countries with the first hospital starting with KMC between 1999 and 2008 (Malawi 1999; Uganda 2002; Rwanda 2007; Mali 2008). Similar facilitating factors and challenges to KMC implementation were identified in all four countries and variation was observed in the quality of KMC implementation between facilities and across countries. Details of key findings from the facility-assessment questionnaire are provided in an online supplementary file (Additional file 2).

### Country progress with the implementation of facility-based KMC

Two of the four countries – Malawi and Rwanda – have embarked in a more focused fashion on scaling up KMC in the past five or six years, using a “big bang” [17] approach. Small scale-up projects were followed by a countrywide initiative, targeting all hospitals and community health centres in Malawi and all district hospitals in Rwanda at the same time as part of newborn care or KMC-specific projects. In Mali and Uganda there was more of a staggered approach linked to available funding, support and local administrator or provider demand for services.

The expansion of KMC services in Malawi and Uganda showed a common pattern of introducing an innovative service in a low- and middle-income country [4,21]. In both countries KMC services were available in only one or two hospitals for a fairly long period of time prior to concerted efforts being made to scale up this intervention. Central or teaching hospitals often get acquainted with a new practice such as KMC first, but then seem to lack the capacity to further diffuse the concept and practice. These hospitals may fall directly under the central ministry of health, whereas the rest of the facilities are serviced through district or regional health offices. This distinction leads to confusion and friction with regard to how central hospital staff may be engaged with staff at other levels of the system – an observation made in three of the countries.

In 37 of the 39 health care facilities visited (95%) there was some evidence of KMC practice, with two facilities in Uganda not achieving this level. Twenty-two facilities (56%) had a score on the fourth stage of change (evidence of practice) (Malawi 8; Mali 6; Rwanda 2; Uganda 6). Thirteen facilities (33%) had a score on the fifth stage of change (evidence of routine and integration) (Malawi 6; Rwanda 4; Uganda 3), whereas two facilities (5%) had reached the level of sustainable practice (Rwanda 1; Mali 1). Individual facility scores out of a potential 30 points ranged between 10.34 and 20.07 in Malawi, between 10.09 and 24.57 in Mali, between 13.19 and 24.05 in Rwanda and between 8.28 and 21.72 in Uganda.

The three facilities with a longer history of KMC in Malawi struggled to maintain routine practices as a result of resource constraints when donor funding or support had run out. Other facilities also reported too many staff rotations and insufficient orientation of new staff. This could have been aggravated by the absence of guidelines and protocols at facility level and because of the magnitude of the countrywide KMC scale-up program to include all health facilities in the country over a short period of time.

Rwandan facilities fared better with implementation progress compared to facilities in Uganda and Mali, despite the comparatively recent initiation of services. One facility had been developed as a centre of excellence for newborn care in 2007. Furthermore, Rwanda had just completed a successful upgrading project in neonatal care in which KMC had been included. Of importance is that the ministry of health took ownership and KMC supervision was included in the supervision of neonatal services at national level.

In Mali one facility served as a centre of excellence, with three KMC champions and a dedicated multidisciplinary team. All KMC training, mainly for hospitals in the south of the country, had been coordinated centrally from this centre. Other facilities fared less well; inter alia as a result of lack of resources to conduct supportive supervision over very long distances, difficulties in buy in experienced as a result of the decentralization of services, and the high mobility of KMC-trained doctors who left the remaining trained nurses behind without the necessary clinical support.

Implementation of KMC services in Uganda appears to have been less systematic and limited in the main to a number of districts in the southern, more populous, part of the country. Implementation was also linked with different projects and approaches in which KMC was more theoretical than practical, possibly with some trainers not having sufficient practical experience in KMC. One facility with a more advanced level of KMC services had additional resources and an energetic KMC leader.

### Facilitators of and barriers to the implementation of KMC services

Similar facilitators and barriers emerged from the stakeholder meetings and interviews and the information gleaned during facility visits. Factors that contribute to the strength of KMC implementation include: training and orientation; supportive supervision; integrating KMC into quality improvement; continuity of care; involvement of and support from government, district, and institutional managers; and client-oriented care. Table 4 provides a key-word summary of the main lessons learned.

**Table 4:** Key lessons regarding the institutionalization of KMC

**Theme**	**Facilitating factors**	**Challenges**
Training and orientation	● Pre-service curricula include KMC	● Lack of clarity of what transpires during training
	● In-service training	● Trainers lack knowledge, skills and experience
		● Non-optimal workplace implementation of KMC
Supportive supervision	● Project-driven interventions bring additional resources for supervision	● Supervision not sustained because of
		- staff workload
		- lack of transport
		- distances
		- decentralization
Integrating KMC into quality improvement	● Use of KMC registers	● No standardized reporting on KMC required at a higher level
	● Inclusion of KMC in mortality and morbidity review meetings	
		● Available data aggregations not used
		● Poor quality of record keeping
		● Recommendations from review meetings not followed up
Continuity of care beyond the facility	● KMC included in antenatal care	● KMC not included in antenatal care
	● Adequate follow-up system for KMC babies	● Poor follow-up of KMC babies due to:
	● Use of community health workers to encourage caregivers to go for follow-up	- poverty
		- travel distances
Governmental and institutional support	● Existence of national KMC policy documents or guidelines	● Unavailability of guideline documents at facility level
	● KMC champions at different levels in the health system	
	● Support from district and facility management	
Client-oriented care	● Promotion of companions in the care of mother and baby	● Low uptake in the use of maternal and newborn services
		● Cultural beliefs (e.g. baby should be carried on the back)

#### Training and orientation

In all four countries facility informants and stakeholders reported that KMC was included in pre-service curricula of nursing-, medical- and other clinical staff. Although national curricula appeared to be standardized, no one had any materials or documentation in this regard at their disposal or information on exactly what students were exposed to and how. Informants from 13 hospitals involved in practical student training had the impression that students had not yet been exposed to KMC when arriving for practical training. In Rwanda four hospitals indicated that some students had an idea of KMC or had heard about KMC, whereas in Malawi informants in one hospital expressed the view that nursing students had more theoretical background on KMC than medical students (doctors and/or clinical officers).

In the case of in-service training, especially where KMC was part of a more comprehensive newborn-care training package, some master trainers were reported as having insufficient personal experience in KMC practice. Some of those health workers sent for training were also reported as not sharing or being unable to share their new knowledge and skills on return to their workplace; and the assessors had the impression that some were not very knowledgeable about KMC. Only 16 (41%) of the 39 participating facilities reported an orientation program in KMC for new staff.

Staff uncertainty about certain aspects of KMC practice could be attributed to insufficient depth of the in-service orientation and training they had received and a lack of experience in caring for preterm infants in low-caseload facilities. In facilities with sufficient space for practising KMC continuously, the assessors had the impression that not all health workers understood the importance of encouraging mothers and caregivers to practice continuous skin-to-skin contact for keeping infants warm. Seventeen facilities (44%) claimed that they promoted intermittent KMC for babies not yet admitted to continuous KMC or where there was no space for rooming-in with their mothers. This was, however, impossible to verify, as no systematic patient records that included KMC had been kept and babies could only be observed in intermittent KMC in five facilities. The majority of participating facilities could show a feeding aid for calculating expressed breast-milk volumes for LBW infants (n = 32; 82%). In Rwanda there were standardized newborn protocols that included the management of KMC [22], but in the other countries few facilities had job aids or protocols in place to guide the rest of KMC practice, except for some training documents available in 13 hospitals (mainly referring to admission and discharge criteria). Malawi and Rwanda had a separate national KMC guidelines document [9,10], which was only available in one hospital in Rwanda.

#### Supportive supervision

In three of the countries the scale up of KMC was linked to a donor project with built-in supervisory activities, often provided by special project staff. Some informants provided anecdotal comments on the deterioration in quality of services after the end of a project, for example: “Almost everything has faded*”*. Health system constraints also contributed to the absence of supervision. These include staff workload, lack of transport, the distance between the district office and health facilities, and internal conflict between different health structures or authorities as a result of decentralization policies.

#### Integrating KMC into quality improvement

We did not probe whether facilities were already conducting regular meetings to review causes of maternal and perinatal mortality and morbidity. For those few facilities in our study where informants incidentally reported these meetings they appeared to be a ‘doctor’s thing’, without much involvement from the nursing staff; i.e. those most involved in supporting KMC services. It was also not clear as to whether the outcomes and recommendations emanating from these meetings were fed into broader quality-improvement processes in the facility.

The quality and completeness of recordkeeping and documentation were highly variable across sites. Although 24 facilities (62%) were able to provide a record review with aggregated data pertaining to KMC taken from general or special KMC registers, the accuracy was often questionable. No facility recorded intermittent and continuous KMC data separately and in two countries some facilities conflated KMC data with the general LBW data that was routinely collected. Where the introduction of KMC was part of a project’s data collection, processing seemed to deteriorate after the end of the project. Staff did not know what to do with their data or did not see a reason for continuing with the effort. In one country facilities were supposed to send regular reports with more detailed information to a central office at the ministry of health, but it was unclear if and how these reports were used to monitor KMC quality or scale up. In the other three countries only seven facilities regularly reported on KMC-specific activities and statistics to a higher level of in-facility management.

#### Ensuring continuity of care beyond the facility providing KMC services

The continuity of care from pregnancy to postnatal care by health workers “proficient and dedicated to KMC” [23] has been highlighted in the literature as important for the promotion of infant well-being. Ten of the 39 participating facilities (26%) were aware of some KMC education taking place during antenatal care in primary health care clinics and, where applicable, at the antenatal clinic in their own facility. Ensuring routine follow-up after discharge from health facilities was reported to pose many challenges in all four countries, especially as a result of poverty and distances. Weak follow-up arrangements such as lack of services close to the communities where women reside were a major barrier to the successful implementation of KMC in all countries. Only 16 participating facilities (41%) could provide good evidence of a follow-up system to review LBW infants regularly until they reached a weight of about 2500 g. Six central or regional hospitals provided specialized follow up until the age of 1 to 2 years, whereas the rest of the facilities (85%) did not have access to a mechanism for referring infants to an appropriate, higher level of follow-up care. Although not probed in depth in this study, there was some evidence of efforts to systematically provide a better continuum of care for newborns by involving community health workers also trained in KMC (e.g. health surveillance assistants in Malawi and *agents de santé maternelle* in Rwanda). At the time of the study, home visits for all LBW infants were not yet institutionalized in any of the countries.

#### Governmental and institutional support for KMC implementation

In all four countries there was some form of buy in and support at the national government level for the implementation of KMC in the form of policy documents that included KMC as a priority in the ambit of newborn care [9,24-28]. Resource allocations from ministries of health for KMC scale up were, however, limited. Despite the shortcomings at district and facility levels discussed above, there were also positive forces facilitating the implementation of KMC. The role of KMC champion was not limited, but individuals at different levels and across cadres assumed leadership. Overall, staff appeared to be enthusiastic about KMC services. The support of senior management at district and facility level – psychological, budgetary, and in-kind – was perceived to play a role in staff motivation and the ability of facilities to move forward with KMC.

#### Client-oriented care

Informants in all four countries reported that KMC was well accepted by mothers and families during their infant’s hospitalization. In African countries allowing a patient to have a companion or ‘guardian’ (usually a family member) to help with the care of the patient while hospitalized is a widespread practice [29]. Twenty-nine facilities (74%) allowed one companion to assist with the care of the mother and infant, with 12 of these (41%) reporting a policy of unrestricted access to the mother and infant any time of the day. These companions were reported to assist with the practice of KMC in most facilities, if the rationale for the method had been adequately explained to them and the mothers. Facilities that did not allow a companion outside visiting hours gave reasons such as the prevention of infection where the space is small and the negative influence of some companions on mothers’ willingness to practice KMC. The novelty of KMC and the fact that the mother carries the preterm infant on her front instead of on her back were also reported as hampering factors for continuing KMC at home after discharge [30]. In two countries babies were observed in KMC in only 29 and 36 per cent of facilities, respectively, and it seems as though the use of maternity services was also low, with little demand for in-patient care.

## Discussion

To the best of our knowledge, this is the first multi-country assessment of the institutionalization status of facility-based KMC services. The implementation of complex health interventions, such as KMC, cannot be compared statistically across four different countries with different contexts, different socio-political histories and economic policies, and widely variable basic development indicators. Therefore the focus of this assessment was on lessons learned and the identification of common barriers and facilitators to KMC implementation across countries (Table 4). The findings of this study are helpful to understand the mechanisms behind uptake and sustainability of KMC services and how strengths can be optimized for further scale up KMC.

Harnessing the resources, infrastructure and capacity of existing programs in maternal, newborn and child health for promoting KMC scale up is an approach that warrants consideration. In a KMC scale-up initiative in Ghana it was found that hospitals that had been accredited with baby-friendly status in the five years prior to the KMC interventions scored significantly higher on implementation progress than the rest of the participating hospitals [18]. Although KMC is linked to activities related to the Baby-friendly Hospital Initiative in countries like South Africa [31], linking to and building on other newborn care initiatives such as Essential Newborn Care, Helping Babies Breathe and the management of basic and comprehensive emergency obstetric and newborn care (BeMONC and CeMONC), could assist with KMC scale up in other countries. In settings where KMC has not yet been initiated, KMC could be used as entry point to improve general newborn services or the “pedestal approach” [18] where KMC is lifted out for special attention could be followed in order to enhance integration into maternal and newborn care services in general.

Not much has been published on the way in which KMC is integrated into pre-service curricula. A study on education and training in the implementation of KMC refers to the academic pathway of KMC implementation where research results are disseminated through teaching [32]. This was the pathway followed in Malawi and Uganda where it took nearly 10 years before KMC could be spread beyond central facilities that were either teaching hospitals or other hospitals with a champion driving a KMC agenda. The variations in health workers’ willingness and ability to further provide in-service KMC training are often a consequence of the train-the-trainer- or cascade approach to disseminating new practices, which is susceptible to a dilution effect [18]. Health worker uncertainty and institutional cultures of verbal transmission of information and practice guidelines may explain the difficulties in some facilities to institutionalize the use of regularly documented information and to develop or use written protocols and standard operating procedures.

An often discussed topic is the difficulties around translating official policy into change in health worker behaviour at grass-roots level, especially where health workers are aware of the value of a particular intervention [6,33]. Embedding practices – initially driven as interventions funded in a project mode – into the existing health system when a project ends or when funding is cut remains problematic [34,35]. One category of facilitators of KMC implementation is opinion leaders, a category that includes people in leadership and management positions at all levels of the health system and the so-called grass-roots “champions” that make KMC work [15,16,32,36]. In our study the support from managers, especially at the district and facility levels, was also found to be a facilitating factor for implementing KMC. A report on a project designed to strengthen supervision in an Integrated Management of Childhood Illness (IMCI) program in Benin refers to “the rise and fall of supervision” [37], concluding that obstacles existed at multiple levels of the health system and that support from leaders and managers in making supervision a priority is essential. Extrapolating from the Benin study and our own findings it appears that important prerequisites for scaling up KMC include sufficient buy in and support at all levels, and sufficient knowledge, skills, time and resources for health workers who have to provide the supervision. Institutionalizing supportive supervision mechanisms for maternal and newborn care, which includes support for KMC, still remains an elusive ideal in many countries [38].

Audit-and-feedback is considered one of the backbones for changing health worker behaviour that would lead to improvement in patient care and better health outcomes for patients [39]. In our study the few facilities that mentioned conducting regular review meetings did not seem to contribute to the creation of internal reporting mechanisms on the progress of KMC implementation. A key barrier to the continued collection and use of KMC data is that KMC scale-up programs are mostly project driven and not part of existing national health management information systems. Having KMC indicators integrated into district and national health management information systems and a routine measure of progress made with implementation could be a way of ensuring continuity and continuation of data collection and use after a project period ends. To do this, health workers as KMC “data producers” (who may even capture the data electronically) need support in how they can become “data users” [40]. To this end, they should be engaged with active and regular feedback on how their data and the summary reports could be or have been used to improve services and how they can improve the quality of their data.

Client-oriented factors outside of the control of the facility providing KMC services have also been reported in other KMC scale-up studies [18,36]. Low uptake of KMC services in two of the countries in our study could possibly be linked to high percentages of home deliveries, lack of awareness of the availability of special care for LBW babies, and a lack of incentives for admitted mothers in the form of provision of free services, regular in-facility meals, or support for companions for mothers. Low uptake may also be linked to travelling distance to the nearest health care facility for both in-patient and follow-up services.

The health system’s capacity to follow up preterm infants after discharge is probably the greatest challenge for low- and middle-income countries [6,36,41-43]. In a Malawian study only 54% of KMC infants completed their review schedule at the hospital [41]. A study in Mali found that non-compliance with periodic visits was one of the major difficulties after discharge [44]. In our study poverty and distance were also reported as factors influencing the ability of a caregiver to bring an infant back for regular follow-up care.

The role of companions as persons to act as a bridge from facility-based- to home-based KMC has, to our knowledge, not yet been the subject of systematic investigation. Reasons for not continuing with KMC after discharge from health facilities need more investigation, as well as interventions that would promote increased compliance with KMC after discharge. Achieving quality practice of KMC in the community, either after facility discharge or initiated at home, is currently a topical issue in newborn care [8]. Using mothers who have successfully ‘graduated’ from KMC to provide peer support and mentoring could be a potential strategy to consider.

This study has several limitations. The convenience sample of facilities means that the results are not generalizable to the remainder of the facilities in the countries surveyed. The 95 per cent of facilities demonstrating some evidence of KMC practice is more likely to reflect the best case scenario. The methodology used and the resulting implementation scores reflect a single ‘snapshot’ and does not track progress over time. While some mothers and caregivers were informally interviewed, the study was not designed to examine issues around demand for KMC services and this remains an important area for further research.

## Conclusion

Scaling up KMC throughout the health system is a complex health intervention and, while worthwhile on many counts, is challenging. Not only does it have to become institutionalized, it also has to be integrated into the continuum of maternal, newborn, and child care. Engaging the ministry of health and other implementing partners from the start in the process of garnering support for KMC assists in mobilizing buy in and resources and ensuring KMC is on district agendas and budgets when scale up commences.

In order to maintain high-quality services, monitoring and supportive supervision should be integrated into existing systems, without overburdening health workers with additional work. Furthermore, the data collected from KMC-related activities might enhance the quality of care in other national perinatal health foci. The integration of KMC into pre-service training with appropriate practical training will ensure health workers enter the field with the requisite knowledge and skills to sustain this important package of services. Finally, KMC at its core is a family-friendly intervention that supports the mother-infant pair from the outset. Involving community members and families in scale up could ensure that this best-practice care is championed from the home to the health facility and back.

## Competing interests

The authors declare that they have no competing interests.

## Authors’ contributions

A-MB, KK, SA and JJ take responsibility for the content of the study, from inception to final publication. NG, BR, RT and EvR contributed to the overall design and interpretation across countries. All other authors were involved in the design and execution of the study in a specific country: RLig and RLuh in Malawi; MK and MS in Mali; BM, FN, FS and JZ in Rwanda; PA, KD and NHS in Uganda. A-MB and KK wrote the first draft of the paper and all other authors contributed to the revision of the manuscript and approved the final manuscript.

## Pre-publication history

The pre-publication history for this paper can be accessed here:

http://www.biomedcentral.com/1472-6963/14/293/prepub

## Supplementary Material

Additional file 1 Kangaroo mother care progress monitoring tool. Click here for file

Additional file 2 Summary of key findings by country. Click here for file
