# Supplementary material for: Implementing facility-based kangaroo mother care services: lessons from a multi-country study in Africa
Source: BMC Health Serv Res. 2014 Jul 8;14:293. doi: 10.1186/1472-6963-14-293 (PMC4104737; doi:10.1186/1472-6963-14-293)
Supplement: Additional file 1 — Kangaroo mother care progress monitoring tool. [file 1472-6963-14-293-S1.pdf]

KANGAROO MOTHER CARE PROGRESS MONITORING TOOL

Use of the instrument / tool:

- This instrument can be used for scoring KMC implementation in health care facilities providing maternity services.
- It can be used to score health care facilities before the implementation of KMC to get an impression of the situation at each facility.
- The questions marked with an asterisk (★) should always be answered, even if the health care facility does not practise KMC.
- The main use of the tool is to assess progress with implementation of KMC after 6-12 months after the launch of the project or the introductory workshop to key health care workers.
- The tool could also be used for one or two years after implementation to assess progress and the potential for sustainability.

Guidelines for monitors / assessors:

- Obtain permission from the facility director to visit the KMC unit, take photographs of hospital staff (after receiving their verbal permission), review records and conduct a key informant interview to complete this tool.
- Attach baseline data to this form (if available).
- Request copies of all written documents related to KMC. If copies are not available, ask for permission to photograph the documents for record purposes.
- Ask for photocopies or samples of forms, registers and relevant material. If copies are not available, ask for permission to photograph the documents for record purposes. Be sensitive to ethical issues and patient privacy. Do not photograph records with patient names on or cover the names before taking the picture.
- Use a written consent form for each mother and/or child to be photographed. Pictures may only be used for reporting on the project and for educational purposes, but not for commercial purposes.
- Mark each of the documents you take away with a date and the name of the hospital or health centre, where applicable.
- Each monitor/assessor fills in his/her own checklist and the results are compared and consolidated afterwards on one checklist, which is then marked as “FINAL”.

Instructions:

- Tick or cross only applicable boxes.
- Complete the **“comments”** and **“observations”** sections if something important or striking is mentioned or observed that may be informative to understanding a particular phenomenon or situation.
- Where possible, complete **“specify”**, **“describe”**, **“explain”** and **“elaborate”** where the associated response is ticked.

Definitions:

- Health care facility: Hospital, community health centre, midwife obstetric unit (MOU) or any other health care facility providing maternity services; also referred to as “the/your facility”
- Neonatal unit: Ward that has at least a (heated) room where babies are cared for in cribs and/or incubators (nursery / high care); it can also comprise a neonatal intensive care section (NICU)
- KMC space/ward: Separate room or area in another ward allocated for mothers and babies in KMC
- Continuous KMC: Baby is carried in the KMC position ± 24 hours per day
- Intermittent KMC: Baby is placed in the KMC position at least once or twice per day for at least 30-60 minutes
- Sporadic KMC: Baby is placed in KMC position less than once per day

Name of progress monitor / assessor: ………………………………….. Date: …………...……………

**KEY**:	★ = Questions for health facilities without KMC<br>I = Complete during interview<br>O = Complete during walk through (observation in ward)<br>P = Assessor completes on his/her own or in team without asking any question (Question 4.3 and questions under 17) (Team reaches consensus on these at the end)<br>® = Items appearing on the report form<br>Pres = Information requested for facility presentation (use interview and observation to verify the information)

OBSERVATIONS AND QUESTIONS TO ASK HEALTH WORKERS

★1 HEALTH CARE FACILITY

★® Pres I 1.1 Region: …………………………………..District: ………………………………………

★® Pres I 1.2 Name of facility (hospital / health centre): ……………………………………………

★ Pres I 1.3 Name of KMC coordinator *(If there is no KMC coordinator, write the name of the nursing manager of the maternity or neonatal ward)*:

…………………......…………………………… Designation: ……...………………………………

★ Pres I 1.4 Other informant/s:

**Name**	**Designation**
…………………………………………………….	…………………………………………………….
…………………………………………………….	…………………………………………………….
…………………………………………………….	…………………………………………………….
…………………………………………………….	…………………………………………………….

★ I 1.5 Level of facility:

Community health centre / clinic / MOU	☐	
Level 1 (District hospital)	☐	
Level 2 (Regional hospital)	☐	
Level 3 (Central / Tertiary / Teaching hospital)	☐	
Other (specify)	☐	……...…………………………………...……

★® I 1.6 Does your facility have baby-friendly status? Yes ☐ No ☐ Unsure ☐

1.6.1 If Yes, when did it get its status? ……………………………….……………….………….

1.6.2 If No, are you planning to become baby-friendly?

Yes ☐ No ☐ Unsure ☐

1.6.2.1 Comments: …………………………………………………………………………

…………………………………………………………………………………….

★2 NEONATAL AND KANGAROO MOTHER CARE

★ I 2.1 Types of neonatal care available: *(Mark as many as applicable)*

- Intensive care ☐
- Incubators (used and unused) ☐
- Warm cribs ☐
- Ordinary cribs in a heated room ☐
- Ordinary cribs in a non-heated room ☐
- Other (specify) ☐ ……………………………………………………………

★ I 2.2 If intensive care is available, where is it done?

- Neonatal intensive care unit (NICU) ☐
- General intensive care unit (ICU) of hospital ☐
- Other (specify) ☐ …………………………………………………
- Not applicable ☐

★ I 2.3 If there are incubators in the neonatal unit:

2.3.1 How many are there (used and unused)? …………………..

2.3.2 How many are in use? …………………..

2.3.3 If none or only a few are in use, what are the reasons? ……………………………………..

……………………………………………………………………………………………

★3 SKIN-TO-SKIN PRACTICES

★ I 3.1 How is a baby cared for in the first hour after birth in this facility? Could you explain the steps and procedures of what happens to the baby? *(Let the informant/s talk freely first and make notes)*

……………………………………………………………………………………………..

……………………………………………………………………………………………..

3.3.1 Skin-to-skin contact between mother and baby mentioned spontaneously?

Yes ☐ No ☐ ***➔ If Yes, go to Question 3.2***

3.3.1.1 If No: Are *any* babies placed in skin-to-skin contact with their mothers during the first hour after birth?

Yes ☐ No ☐ Unsure ☐

(a) If Yes, which babies are placed in a skin-to-skin position?

……………………………………………………………………………………

® I O 3.2 Type(s) of kangaroo mother care practised:
(Mark as many as applicable; specify further as needed)

No KMC practised	☐	➔ ***Go to Question 4.4***
Intermittent KMC	☐	……………………………………………………………………………
Continuous KMC	☐	……………………………………………………………………………
Sporadic KMC	☐	……………………………………………………………………………
Other (specify)	☐	……………………………………………………………………………

4 HISTORY OF KMC IMPLEMENTATION

® I 4.1 When was KMC started? ……………………………………………………………………….

I 4.2 Tell us more about the process that was followed.
(Take notes and probe for the points below, if not mentioned)

Don't know ☐ ➔ ***Go to Question 4. 3***

……………………………………………………………………………………………………..

……………………………………………………………………………………………………..

……………………………………………………………………………………………………..

4.2.1 Was there a specific occasion or meeting where the decision to implement KMC was taken?

Yes ☐ No ☐ Unsure ☐

4.2.2 Approximate date: ………………………………………………………………………..

4.2.3 What was the occasion? ……………………………………………………………………...

4.2.4 Are there *written* minutes or a report of the decision?

Yes ☐ No ☐ Unsure ☐

(If Yes, ask if it would be possible to see a copy.)

4.2.4.1 Copy of written document seen Yes ☐ No ☐

(If Yes, get a copy or take a picture)

4.2.5 Who was involved in the decision-making process? ……………………………………

………………………………………………………………………………………………..

P 4.3 Monitor's / Assessor's impression of recall of history of implementation

Good recall ☐ Some recall ☐ No recall ☐

➔ ***Go to Question 4.5***

★ I 4.4 *(If KMC is not implemented yet)* Has a formal decision for KMC implementation been made yet?

Yes ☐ No ☐ Unsure ☐

4.4.1 If Yes, describe: ……………………………………………………………………………

……………………………………………………………………………………………

……………………………………………………………………………………………

➔ Go to Question 9

I 4.5 Did the facility do a baseline survey on the neonatal mortality and / or morbidity rates before starting with KMC?

Yes ☐ No ☐ Unsure ☐

➔ If No or Unsure, go to Question 4.6

4.5.1 If Yes, describe: ……………………………………………………………………………

……………………………………………………………………………………………

(Ask if a copy is readily available to see.)

4.5.1.1 Copy of survey results seen Yes ☐ No ☐

➔ If No, go to Question 4.6

4.5.1.2 Copy of survey results received Yes ☐ No ☐

(If No, take a picture of cover page)

I 4.6 Did the chief executive officer of the hospital / the district health manager / head of facility sign a commitment or undertaking or agreement that s/he would ensure that KMC is implemented in the hospital?

Yes ☐ No ☐ Unsure ☐

4.6.1If Yes, specify further (if necessary): ……………………………………………………

……………………………………………………………………………………………

5 INVOLVEMENT OF ROLE-PLAYERS

® I 5.1 Who are the people who were initially involved in starting KMC?
(Let informant/s first talk freely; take notes and probe for the persons below, if they are not mentioned specifically)

……………………………………………………………………………………………

……………………………………………………………………………………………

® I 5.2 What kind of support did you get from the following people:

District health manager / CEO / superintendent	…………………………………………………………………………
Matron / Nursing service manager	…………………………………………………………………………
Unit manager (neonatal unit or maternity)	…………………………………………………………………………
Clinician (doctor / medical or clinical officer)	…………………………………………………………………………

I 5.3 Are there other people in the hospital from whom you got special support?

Yes ☐ No ☐ Unsure ☐

5.3.1 If Yes, who and what kind of support? …………………………………………………………

…………………………………………………………………………………………………

6 RESOURCES

® I 6.1 Did you get any allocations from the hospital or district budget to establish your KMC facility?

Yes ☐ No ☐ Unsure ☐

6.1.1 If Yes, what was the nature of the allocation / what was the money used for?

…………………………………………………………………………………………………

…………………………………………………………………………………………………

® I 6.2 Did you have other sponsors? Yes ☐ No ☐ Unsure ☐

(Also probe for donations in the form of material, wraps, caps, furniture, paint, labour [e.g. for making the space pretty] etc from churches, or other community, volunteer or religious groupings, or individuals in the community)

6.2.1 If Yes:

Name of sponsor	Nature of contribution
………………………………………	………………………………………………
………………………………………	………………………………………………

7 KANGAROO MOTHER CARE SPACE: CONTINUOUS KMC

(Please note: complete this section ONLY if the hospital of health centre has a room or space where mothers and their babies room-in for 24 hours per day and where the baby is with the mother all the time in the KMC position)

® I O 7.1 Is there a ward or special area in another ward allocated for KMC?

Yes ☐ No ☐ ➔ ***If No, go to Question 8***

7.1.1 If Yes, what is nature of the space available?

Separate ward / unit ☐
Space / Corner in another ward ☐
Other (specify) ☐ ………………………………………………………

7.1.1.1 If space or a corner in another ward is used, which ward?
7.1.1.2

Postnatal ward ☐
Paediatric ward ☐
Other (specify) ☐ ……………………………………………………………

® I O 7.2 Number of KMC beds: …………………..

® I O 7.3 Number of mother-baby pairs enrolled for KMC at the moment (i.e. how many KMC beds occupied?):

…………………..

® O 7.4 Number of mothers having babies in KMC position at time of walk-through: ……………..
(Must have been observed in person)

O 7.5 *If there is no mother or baby in KMC, ask for records of the last few babies that went through KMC.*

Records could be provided Yes ☐ No ☐

7.5.1 If Yes, is there any evidence of KMC practised in records?

Yes ☐ No ☐

7.5.1.1 If Yes, what? ………………………………………………………………………

7.6 Are there any cribs in the KMC space / ward? Yes ☐ No ☐

I O 7.7 How are babies tied in the KMC position? *(Mark as many as applicable)*

Local cloth (e.g. *chitenje*) ☐
Special triangle and blouse ☐
Kalafong *thari* ☐
Square cloth with band ☐
Draw sheets ☐
Towels ☐
Other (specify) ☐ ……….……………………………………………………..
……….……………………………………………………..

® I O 7.8 Which of the following equipment or facilities are available in the KMC space?

Low beds	☐	
Head rests or pillows for mothers to lean against	☐	
Chairs	☐	Comfortable? …………………………..
Other (specify)	☐	……………………………………………
		……………………………………………

® I 7.9 For how many hours per day are the babies in the KMC position? …………….. hours

7.9.1 When are the babies *not* in the KMC position? ……………………………………….....

…………………………………………………………………………………………………...

® I O 7.10 Who decides when a baby is ready to go to (intermittent or continuous) KMC? *(Let informant talk first before ticking or probing) (Mark as many as applicable)*

……..

Routine for mother-baby dyads	☐	
Clinicians (clinical or medical officers)	☐	
Nurses	☐	
Patient attendants	☐	
Mother's request	☐	
Other (specify)	☐	………………………………………………………

I 7.11 Which mothers or babies are *excluded* from going to KMC before discharge from the facility? *(Mark as many as applicable; specify further as needed) (Let informant/s first talk freely)*

……

None	☐	……………………………………………..
HIV+ mothers	☐	……………………………………………..
Mothers with another infectious disease	☐	……………………………………………..
Babies born outside the facility / before arrival	☐	……………………………………………..
Babies below a certain weight (specify)	☐	……………………………………………..
Babies above a certain weight (specify)	☐	……………………………………………..
Other (specify)	☐	……………………………………………..

® I O 7.12 What is the policy on the movement of mothers *with their babies in the KMC position*? (E.g. Do they walk around? Are they allowed to leave the ward? Where are they allowed to go and under what conditions? When do they leave their babies behind and when not?)

………………………………………………………………………………………………………..

………………………………………………………………………………………………………..

I O 7.13 Are mothers allowed to have a guardian or companion (e.g. grandmother, sister, baby's father) to assist her with KMC?

Yes ☐ No ☐ ➔ ***If No, go to Question 8***

7.13.1 If Yes, please describe the following:

7.13.1.1 Tasks of this support person: ……………………………………………………..

…………………………………………………………………………………………

7.13.1.2 Times of the day this person is allowed to be with the mother:

…………………………………………………………………………………………

7.13.1.3 Any other information on these support persons that may be important for the assessors to know:

…………………………………………………………………………………………

8 NEONATAL UNIT OR NURSERY: INTERMITTENT KMC

➔ ***If there is no nursery or no KMC is practised in the facility, go to Question 9***

I O 8.1 Is intermittent KMC practised in the nursery or neonatal unit?

Yes ☐ No ☐ Sometimes ☐ ➔ ***If No, go to Question 9***

8.2 If Yes or Sometimes, describe when (i.e. criteria for eligibility): …………………………………

……………………………………………………………………………………………………..

I O 8.3 If Yes

® 8.3.1 Are there fixed times of the days that mothers practise intermittent KMC?

Yes ☐ No ☐ Sometimes ☐ Unsure ☐

8.3.1.1 If Yes or Sometimes, please describe: …………………………………………..

………………………………………………………………………………………..

8.3.2 Is there a written programme available for the times when KMC is supposed to be practised?

Yes ☐ No ☐ Unsure ☐

(If Yes, try to get a copy)

8.3.3 Are the times / occasions when it is recorded somewhere when a baby gets intermittent KMC?

Yes ☐ No ☐ Unsure ☐

8.3.3.1 If Yes, describe: ……………………………………………………………………..

® I O 8.4 Number of babies currently in nursery: …………………..

® O 8.5 Number of babies doing intermittent KMC: …………………..

Observed	☐	Number: ………………………………………………………………..
Verified from records	☐	Number: ………………………………………………………………..
Verified from mothers	☐	Specify: ………………………………………………………………..

® O 8.6 *If there is no baby in KMC, request the records of the last baby that received KMC.*

Records could be provided Yes ☐ No ☐

8.6.1 If Yes, is there any evidence of KMC practised in records?

Yes ☐ No ☐

8.6.1.1 If Yes, what? ……………………………………………………………………

® I O 8.7 When is a baby eligible to start with intermittent KMC? (What criteria do you use to decide if a baby can start intermittent KMC?)

………………………………………………………………………………………………….

………………………………………………………………………………………………….

® I O 8.8 Who decides when a baby is ready to start with intermittent KMC? *(Let informant/s talk first and make notes before ticking or probing) (Mark as many as applicable)*

………………………………………………………………………………………………….

Clinicians (clinical or medical officers)	☐	
Nurses	☐	
Other (specify)	☐	…………………………………………………………

8.9 Where do mothers sit while practising KMC? ……………………………………………………..

………………………………………………………………………………………………….

★9 FEEDING AND WEIGHT MONITORING

★ I 9.1 Is there a place near or at the hospital where the mothers can stay / lodge while their babies are in the neonatal unit / nursery (before they start with KMC if KMC is practised)?

Yes ☐ No ☐ Unsure ☐ Not applicable+ ☐

(+E.g. community health centre)

➔ If No or Unsure, go to Question 9.1.2
➔ If Not applicable, go to Question 9.2

9.1.1 If Yes:

9.1.1.1 Describe where the mothers stay: ………………………………………………

…………………………………………………………………………………………

9.1.1.2 How far is the place from the neonatal unit / nursery? ……………………………..

…………………………………………………………………………………………

9.1.1.3 Is it possible for mothers to come for *all* feeding sessions at night?

Yes ☐ No ☐ Unsure ☐

➔ **Go to Question 9.2**

9.1.2 If No or Unsure:

9.1.2.1 What are the reasons why mothers don't have a place to stay at the hospital?

…………………………………………………………………………………………

…………………………………………………………………………………………

9.1.2.2 What happens if a mother cannot come for all the feeds?

…………………………………………………………………………………………

…………………………………………………………………………………………

★® O 9.2 Is there a *written* feeding policy or protocol for babies in the neonatal ward / nursery and (where applicable) in the KMC space? *(Get a copy to take along or take a picture)*

Yes ☐ No ☐ Unsure ☐

9.2.1 If Yes, can a copy be provided? Yes ☐ No ☐

★® O 9.3 Are there job aids for feeding available in the neonatal ward / nursery and (where applicable) in the KMC space? *(Get a copy or take a picture)*

Yes ☐ No ☐ Unsure ☐

9.3.1 If Yes, where is/are this/these aid/s kept? *(Mark as many as applicable; comment further as needed)*

In a cupboard ☐ ………………………………………………

In a file at the nurses' station ☐ ………………………………………………

Displayed on the wall ☐ ………………………………………………

Other (specify) ☐ ………………………………………………

★® O 9.4 Feeding records: *(Request to see the records of babies)*

9.4.1 Are there regular recordings of *each* feed for *each* baby?

Yes ☐ No ☐ Unsure ☐

9.4.2 If Yes, what is recorded for each feed? *(Mark as many as applicable)*

Time of feed ☐

Volume of feed (if expressed breast milk and/or formula is used) ☐

Nurse's notes ☐

Clinician's notes (medical or clinical officers) ☐

Other (specify) ☐

………………………………………………………………………………

★ O 9.5 Records of weight:

® 9.5.1 Are all babies weighed regularly?

Yes ☐ No ☐ Unsure ☐

➔ *If No or Unsure, go to Question 9.6*

9.5.2 If Yes:

9.5.2.1 How often are they weighed?

More than once every day ☐
Once every day ☐
Every two days ☐
Twice per week ☐
Once per week ☐
Other ☐ Specify: ……..………..……………………

9.5.2.2 How and where is the weight recorded? *(Observe and let informant/s first talk freely before probing below)*

……………………………………………………………….……………………..….

……………………………………………………………….……………………..….

Special weight book ☐
Ward register ☐
Other ☐ Specify: ………………..…………...………………

★ O 9.6 Scale:

9.6.1 Type of scale:

Manual ☐ Brand name: ……………………………………………………………….
Electronic ☐ Brand name: ……………………………………………………………..
Other ☐ Specify: ……………………………………………………………………

9.6.2 Increments:

5 grams	☐	50 grams	☐
10 grams	☐	100 grams	☐
20 grams	☐	Other (specify)	☐ ……..………………..…………………

➔ *If no KMC is practised in the facility, go to Question 12*

10 RECORDS IN USE FOR KMC INFORMATION

® I O 10.1 What kinds of general records are being used for recording KMC information? *(Mark as many as applicable) (If it is not a standardised record, attach copies or pictures of forms or a few pages of a register/book, with names crossed out)*

- Official register provided by the Ministry / Health Directorate ☐
- Special KMC register or collective record kept for all babies who receive(d) KMC ☐
- Discharge scoring sheet ☐
- KMC daily notes ☐
- Other special form for every single KMC baby (e.g. as part of file) ☐
- Discharge letter *with information on KMC* ☐
- Road to health chart / booklet *with information on KMC* ☐
- Other (specify) ……………………………………………………………………… ☐

® Pres I O 10.2 Can figures be provided on how many babies received *intermittent* KMC in a particular period?

Yes ☐ No ☐ Unsure ☐ Not applicable ☐

➔ If No or Unsure or Not applicable, go to Question 10.3

10.2.1 If Yes:

10.2.1.1 Can it be calculated, how many hours per day a baby received intermittent KMC?

Yes ☐ No ☐ Unsure ☐

10.2.1.2 Can it be calculated, for how many days a baby received intermittent KMC?

Yes ☐ No ☐ Unsure ☐

10.2.1.3 Can a baby's daily weight gain while receiving intermittent KMC be calculated?

Yes ☐ No ☐ Unsure ☐

10.2.2 *Note for monitors: Look very carefully at any statistics and see if you can find any anomalies (e.g. no or fewer babies in KMC some months, evidence of poor record keeping, etc). Discuss your observations with the informant/s and try to find reasons for any anomalies.*

……………………………………………………………………………………………...

……………………………………………………………………………………………...

……………………………………………………………………………………………...

……………………………………………………………………………………………...

® Pres I O 10.3 Can figures be provided on how many babies received *continuous* KMC in a particular period?

Yes ☐ No ☐ Unsure ☐ Not applicable ☐

➔ If No or Unsure or Not applicable, go to Question 10.4

10.3.1 If Yes:

10.3.1.1 Can it be calculated, for how many days a baby received continuous KMC?

Yes ☐ No ☐ Unsure ☐

10.3.1.2 Can a baby's daily weight gain while receiving continuous KMC be calculated?

Yes ☐ No ☐ Unsure ☐

10.3.2 *Note for monitors: Look very carefully at any statistics and see if you can find any anomalies (e.g. no or fewer babies in KMC some months, evidence of poor record keeping, etc). Discuss your observations with the informant/s and try to find reasons for any anomalies.*

…………………………………………………………………………………………….

…………………………………………………………………………………………….

…………………………………………………………………………………………….

…………………………………………………………………………………………….

® I O 10.4 Can audit figures or statistics for *at least one year* be provided containing evidence of sustained KMC practice? *(E.g. how many babies went through KMC, how many babies in each LBW category, average/mean birth weight, average/mean discharge weight, average/mean number of days babies spent in hospital, survival rate [number and percentage])*

10.4.1 For intermittent KMC	Yes	☐	No	☐	Unsure	☐	N/A	☐
10.4.2 For continuous KMC	Yes	☐	No	☐	Unsure	☐	N/A	☐
10.4.3 Only KMC in general	Yes	☐	No	☐	Unsure	☐	N/A	☐

10.5 Are there any statistics on KMC displayed somewhere (e.g. on a wall)?

Yes ☐ No ☐ Unsure ☐

10.5.1 If Yes, describe: ………………………………………………………………………….

® I 10.6 Are there official channels through which KMC is reported to different levels of management on a regular basis?

Yes ☐ No ☐ Unsure ☐

® 10.6.1 If Yes, elaborate: …………………………………………………………………….

…………………………………………………………………………………………….

11 KMC EDUCATION

® I O 11.1 Is there a *written* checklist for all the procedures to go through when a mother and her baby are admitted to the KMC space?

Yes ☐ No ☐ Unsure ☐

(If Yes, get a copy or take a picture)

11.1.1 If yes, elaborate: ……………………………………………………………………………………..

………………………………………………………………………………………………………

® I O 11.2 What written and audiovisual information on KMC is available for mothers? *(Get copies or pictures of each if it is not standard material provided by the government or the implementation project)*

Posters	☐	Which posters? ……………………………………………. ………………………………………………………………
Brochures / Information sheets	☐	Describe: ………………………………………………… ………………………………………………………………
Video/DVD	☐	Describe: ………………………………………………… ………………………………………………………………
Counselling cards	☐	Describe: ………………………………………………… ………………………………………………………………
Other	☐	Specify: …………………………………………………… ………………………………………………………………

® I O 11.3 Is verbal education related to intermittent and/or continuous KMC provided to mothers? *(Let informant/s first talk freely, before probing the points below)*

Yes ☐ No ☐ Unsure ☐

………………………………………………………………………………………………………

……………………………………………………………………………………………………..

11.3.1 If Yes, at what point / when? *(Mark as many as applicable; describe further)*

Antenatal care+	☐	……………………………………………………
During transport to hospital	☐	……………………………………………………
Immediately after birth	☐	……………………………………………………
While baby is in nursery	☐	……………………………………………………
When mother and baby are transferred to / start KMC	☐	……………………………………………………
Other (specify)	☐	…………………………………………………… ……………………………………………………

O *+If KMC is reported to take place at antenatal care, request a visit the antenatal care clinic if possible to get a feeling and sense of what is being done in this regard)*

® I O 11.4 Is there a daily or weekly educational or recreational programme for mothers in KMC?

Yes ☐ No ☐ Unsure ☐

11.4.1 If Yes, describe: *(Include a copy if available or a picture)* ……………………………….

……………………………………………………………………………………………………

★**12 DOCUMENTS**

★O 12.1 What general statements like a vision and mission are visibly displayed in the hospital?

……………………………………………………………………………………………………..

★O 12.2 Are there special vision and mission statements for the neonatal unit / nursery / maternity (under which KMC is practised)?

Yes ☐ No ☐ Unsure ☐

➔ If No or Unsure, go to Question 12.3

® 12.2.1If Yes, do any of these statements mention KMC?

Yes ☐ No ☐ Unsure ☐

12.2.1.1 If Yes, describe *(or include a document or picture as evidence):*

……………………………………………………………………………..

★® O 12.3 Are there any *written* policies, guidelines or protocols regarding the practice of KMC?

Yes ☐ No ☐ Unsure ☐

➔ If No or Unsure, go to Question 13

12.3.1 If Yes:

12.3.1.1 For what type of KMC are the policies, guidelines or protocols meant? *(Mark as many as applicable) (Get copies or pictures)*

Intermittent KMC	☐	………………………………………………………
Continuous KMC	☐	………………………………………………………
Other	☐	Specify: ……………………………………………

12.3.1.2 For which target groups? *(Mark as many as applicable; describe further as needed) (Get copies or pictures)*

General instructions for ward	☐	……………………………………………
For nurses	☐	……………………………………………
For clinicians	☐	……………………………………………
For patient attendants	☐	……………………………………………
Other	☐	Specify: ………………………………
		……………………………………………

12.3.1.3 Where do these guidelines, policies or protocols come from?

- Taken over as is from examples provided during training ☐
- Taken over as is from other institutions' documents ☐
- Adapted from examples provided during training ☐
- Adapted from other institutions' documents ☐
- Original policy/protocol/guidelines developed locally ☐
- Other (specify) ☐

…………………………………………………………………

12.3.1.4 Who drafted or adapted the policy, protocol or guidelines?
(Let informant/s talk first before ticking or probing)

……………………………………………………………………………………..

………………………………………………………………………………………

- One person ☐ ……………………………………………………………
- Group of persons ☐ Specify: ……………………………………………….
  …………………………………………………………

12.3.1.5 Was the draft policy, protocol or guidelines distributed further for comments?

Yes ☐ No ☐ Unsure ☐

(a) If Yes, to whom? ……………………………………………………………

………………………………………………………………………………

★13 REFERRALS, DISCHARGE AND FOLLOW-UP

★® I 13.1 Who decides when a baby is ready for discharge?

- Clinicians (clinical or medical officers) ☐
- Nurses ☐
- Patient attendants ☐
- Mother's request ☐
- Other ☐ Specify: …………………………………………..

★ I 13.2 What criteria are used to decide if a baby is ready to be discharged?
(Let informant/s first talk freely before probing the points below)

……………………………………………..……………………………………….……………

………………………………………….…………………………………………………………..

Baby has reached certain weight	☐	Specify: …………………………………………..
Baby gains weight consistently	☐	Specify: …………………………………………..
Mother is willing to continue KMC	☐	Specify: …………………………………………..
Other	☐	Specify: ………………………………………….. ……………………………………………………..

★® O 13.3 Is a special discharge scoring sheet used to help with the discharge decision?

Yes ☐ No ☐ Unsure ☐

★® I 13.4 Where are the majority of preterm / LBW /KMC babies followed-up after discharge from hospital?

At the hospital where baby has been born	☐	➔ ***Go to Question 13.5***
At hospital nearest to mother's home	☐	➔ ***Go to Question 13.6***
At the nearest community centre / clinic	☐	➔ ***Go to Question 13.6***
Other	☐	Specify: ………………….……………… ………………………………………..…… ➔ ***Go to Question 13.6***

★® I O 13.5 If babies are followed up at the hospital where they have been born:

13.5.1 Where are they followed up?

In the KMC space/ward	☐	
At the nursery/neonatal unit	☐	
At the outpatients department	☐	
Other (specify)	☐	…………………………………………………….
Not applicable	☐	

I O 13.5.2 Are records kept of follow-up visits? *(Take a blank copy or take a picture)*

Yes ☐ No ☐ Unsure ☐

13.5.2.1 If Yes, specify/describe: ……………………………………………………………

13.5.3 Until what weight are they followed up at the hospital? ……………………..

13.5.4 What is the follow-up rate of babies? (What percentage of babies are brought back to the hospital for review?)

…………………………………………………………..………… Not known ☐

13.5.5 What measures are in place to ensure that babies are brought back to the hospital for review?

……………………………………………………………………………………. None ☐

……………………………………………………………………………………………………..

★ I 13.6 What measures of communication are in place to inform a health centre of a baby's discharge from hospital? *(Specify further if necessary)*

None	☐	………………………………………………
Phone call, fax or e-mail to health centre	☐	………………………………………………
Phone call, fax or e-mail to district office	☐	………………………………………………
Referral letter given to mother or guardian	☐	………………………………………………
Other (specify)	☐	………………………………………………

★ I 13.7 Are home visits done? Yes ☐ No ☐ Unsure ☐

13.7.1 If Yes:

13.7.1.1 Are *all* babies visited or *only some*? All ☐ Only some ☐

(a) If Only some, which babies are visited? ……………………………………….

…………………………………………………………………………………………

13.7.1.2 Who does the home visits? *(Describe further where needed)*

Community health workers (CHWs)	☐	………………………………………………
Community-based surveillance volunteers (CBSVs) / Health surveillance assistants (HSAs)	☐	……………………………………………… ………………………………………………
Nurses (community health, public health)	☐	………………………………………………
Community health officers (CHOs)	☐	………………………………………………
Other (specify)	☐	………………………………………………

★® I 13.8 Are babies transported to your hospital or health centre in the skin-to-skin (KMC) position?

Always ☐ Sometimes ☐ Seldom ☐ Never ☐ No experience ☐

★® I 13.9 Are babies transported from your facility to another in the skin-to-skin (KMC) position?

Always ☐ Sometimes ☐ Seldom ☐ Never ☐ No experience ☐

➔ ***If KMC has not yet been implemented in the facility, go to Question 15***

14 STAFF ORIENTATION AND TRAINING

® Pres I 14.1 How many staff members were originally trained in KMC *outside* your facility?

Don't know ☐

**Total:** ………………	**Number**
Managers (e.g. district health offices, matron)	………..
Clinicians (clinical or medical officers)	………..
Nurses / Midwives	………..
Patient attendants	………..
Other (specify) (e.g. cleaners, volunteers)	………..

……………………………………………………….

……….……………………………………………….

Pres I 14.2 What kind of awareness and educational activities did you have in your facility to introduce KMC to staff members?

None ☐

……………………………………………………………………………………………………..……

® Pres I 14.3 How many staff members have up to now been fully trained in KMC *inside* your facility?

Don't know ☐

**Total:** ………………	**Number**
Managers (e.g. district health officers, matrons)	………..
Clinicians (clinical or medical officers)	………..
Nurses / Midwives	………..
Patient attendants	………..
Other (specify) …………………………………..	………..

…………………………………………………….

I 14.4 Number of staff members trained in KMC who still work with KMC: ……………

I 14.5 How often do you get new staff in the maternity or neonatal unit or the community health centre?

……………………………………………………………………………………………………..

® I O 14.6 Is there a special orientation programme for new staff who will work with KMC?

Yes ☐ No ☐ Unsure ☐

14.6.1 If Yes:

14.6.1.1 What is the nature of this orientation? *(Describe further as needed)*

Oral presentation	☐	……………………………………
Written documents to study	☐	……….…………………………
Audiovisuals (e.g. video, CD, DVD)	☐	……….…………………………
Other (specify)	☐	…….………………………………

O 14.6.1.2 What evidence is there of this programme? *(Mark as many as applicable)*

Programme outline available in writing	☐
Notes used in programme available	☐
Health workers confirmed training verbally	☐
Signed in-service or training records	☐
Other (specify)	☐ ………………………………
	………………………………

(Ask to see the evidence. Take a picture of the cover of the file and a few pages inside)

I 14.7 Is there a long-term plan in the hospital or district to get all health workers trained and updated in KMC?

Yes ☐ No ☐ Unsure ☐

14.7.1 If Yes, is this plan *written*? Yes ☐ No ☐ Unsure ☐

(If Yes, ask if it would be possible to see a copy.)

14.7.1.1 Copy of written plan seen Yes ☐ No ☐

(If Yes, get a copy or take a picture)

I 14.8 Is KMC linked with or are there plans to link KMC with other initiatives such as Essential Newborn Care or the Baby-friendly Hospital Initiative?

Yes ☐ No ☐ Unsure ☐

14.8.1 If Yes, with which initiatives?

**Initiative**	**Nature of link**
…………………………………………..	………………………………………………………..
…………………………………………..	………………………………………………………..
……………………………………………	………………………………………………………..

I O 14.9 Do students do practical work in your maternity or neonatal unit?

Yes ☐ Some ☐ No ☐ Unsure ☐

→ *If No or Unsure, go to Question 15.1*

I 14.9.1 If Yes or Some:

14.9.1.1 At which institutions or college do these students receive their theoretical training?

14.9.1.2 Which students do their practical work in your facility?

Nursing ☐
Medical ☐
Nutrition ☐
Other ☐ Specify: ……………………………………………………..……..

I 14.9.1.3 What is your general impression about their training in KMC? *(Mark one answer that reflects your impression the best)*

Generally they have no training in KMC ☐
Some students have an idea of KMC or have heard about KMC ☐
Some students have been exposed to KMC ☐
Generally students know the basics of KMC ☐
Other (specify) ☐

……………………………………………………………………………………

I 14.9.2 Is there a special general orientation programme for students when they arrive at the hospital of health centre?

Yes ☐ No ☐ Unsure ☐

14.9.2.1 If Yes: Is KMC included in the programme?

Yes ☐ No ☐ Unsure ☐

(Ask to see a written copy of a programme if it exists. Take a copy or a picture)

I 14.9.3 Do students get on-the-job training in KMC?

Yes ☐ Sometimes ☐ No ☐ Unsure ☐

14.9.3.1 (*If there are students in the ward when you visit)* Ask students about their training experience in KMC.

……………………………………………………………………………………

……………………………………………………………………………………

……………………………………………………………………………………

……………………………………………………………………………………

★15 STAFF ROTATIONS

★I 15.1 Which nurses rotate between day and night shifts in the maternity or neonatal unit?

All ☐ Some ☐ None ☐

15.1.1 If Some, who does not do day/night shift? ……………………………………………

➔ If the facility is at the health centre level, go to Question 16

★ I 15.2 Are staff members rotated between different wards in the hospital (e.g. between maternity, surgery, male ward, female ward, etc)?

Yes ☐ No ☐ Unsure ☐

➔ If No or Unsure, go to Question 16

15.2.1 If Yes:

15.2.1.1 Are all or only some of the staff members rotated?

All ☐ Some ☐

15.2.1.2 Which of the following cadres are rotated?

Managers (nursing etc) ☐
Clinicians (clinical or medical officers) ☐
Nurses ☐
Patient attendants ☐
Other (specify) ☐ ……………………………………

15.2.1.3 Is there a core of staff in the neonatal unit or maternity ward where KMC is practised that is not rotated to other wards?

Yes ☐ No ☐ Unsure ☐

(a) If Yes, describe: ……………………………………………………………..……..

15.2.1.4 How often do rotations take place?

Every month ☐
Every 3 months ☐
Every 6 months ☐
Every year ☐
Other (specify) ☐ ………………………………………………………………

★16 STRENGTHS AND CHALLENGES

Pres I 16.1 What do you think are the strengths in your facility that facilitated implementation (made implementation easier)?

Not applicable / Not yet implementing KMC ☐ ***➔ Go to Question 16.2***

………………………………………………..…………………………………………………..

………………………………………………………..………………………………………….

★ Pres I 16.2 What are or were the barriers or obstacles to the implementation of KMC?

………………………………………………………..………………………………………….

………………………………………………………..…………………………………………..

QUESTION 17
GENERAL OBSERVATIONS AND IMPRESSIONS

(Instruction for assessors: Reach consensus as a group on Questions 17.1 to 17.4 a well as Question 4.3 after the interview and the observations)

® **P** 17.1 Impressions regarding the intensity of involvement of senior management (superintendent, medical manager, CEO, nursing service manager/matron) in establishing KMC (past or future)

A lot of involvement and/or support (moral, material, etc)	☐
Some involvement and/or support (moral, material, etc)	☐
Neutrality / Little support / Resistance	☐

17.1.1 Comments: …………………..……………………………………………….………..

……………………..…………..……………………………………………….………..

P 17.2 Impressions of mothers' compliance with KMC? (I.e. do they always practise it or not?)

Mothers are diligent in carrying their babies in the KMC position	☐
Mother carry their babies in the KMC position some of the time	☐
Very little of KMC actually practised by mothers	☐
Could not probe well enough	☐
KMC not implemented yet	☐

17.2.1 Comments: …………………..……………………………………………….………..

……………………..…………..……………………………………………….………..

★ **P** 17.3 Impressions of the quality of data captured in records

Excellent	☐
Average (only minimum requirements)	☐
Poor	☐

17.3.1 Comments: …………………..……………………………………………….………..

……………………..…………..……………………………………………….………..

★ **P** 17.4 Impressions regarding the quality of the follow-up system

Well-developed (written proof could be supplied)	☐
Partly developed (no written proof but strong evidence of a well organised system)	☐
Non-existent	☐

17.4.1 Comments: …………………..……………………………………………….………..

……………………..…………..……………………………………………….………..

★ Pres P 17.5 Other comments and observations (e.g. where KMC is practised, observe "well-being" of mothers doing KMC, fixing baby, homeliness of space/ward etc)

…………………………………………………………………………………………………

…………………………………………………………………………………………………

…………………………………………………………………………………………………

…………………………………………………………………………………………………

…………………………………………………………………………………………………

★ P 17.6 Comments for hospital *(Use this as basis for giving immediate feedback to hospital, either verbally or on separate sheet)*

P **17.6.1 GENERAL IMPRESSIONS OF MONITOR/ASSESSOR**
(Organise your comments around the headings below)

General: ………………………………………………………………………………………

…………………………………………………………………………………………………

KMC practice (including feeding) …………………………………………………………

…………………………………………………………………………………………………

Documentation and protocols: ……………………………………………………………

…………………………………………………………………………………………………

Involvement of management (different levels): ………………………………………

…………………………………………………………………………………………………

Other: ……………………………………………………………………………………………

…………………………………………………………………………………………………

P **17.6.2 RECOMMENDATIONS FOR CONSIDERATION**
(Organise your comments around the headings below)

General: ………………………………………………………………………………………

…………………………………………………………………………………………………

KMC practice (including feeding) …………………………………………………………

…………………………………………………………………………………………………

Documentation and protocols: ……………………………………………………………

…………………………………………………………………………………………………

Involvement of management (different levels): ……..…………………..……………………………

……………………………………………………………………………..……………………………

Other: ………………………………………..……..…………………..……………………………

……………………………………………………………………………..……………………………

Pres 18.7 Ideas for policy makers and health authorities

……………………………………………………………………………………………………

……………………………………………………………………………………………………

……………………………………………………………………………………………………

……………………………………………………………………………………………………

……………………………………………………………………………………………………

………………………………………..……… ………………….…………………………

NAME OF MONITOR / ASSESSOR | Signature

…………………….………………………………

Date
