# Supplementary material for: Implementing facility-based kangaroo mother care services: lessons from a multi-country study in Africa
Source: BMC Health Serv Res. 2014 Jul 8;14:293. doi: 10.1186/1472-6963-14-293 (PMC4104737; doi:10.1186/1472-6963-14-293)
Supplement: Additional file 2 — Summary of key findings by country. [file 1472-6963-14-293-S2.pdf]

Supplementary online file

Summary of key findings by country

Topic	Malawi (n=14)	Mali (n=7)	Rwanda (n=7)	Uganda (n=11)
**Facilities targeted for KMC implementation**	• All districts, with focus on district hospitals and community health centres	• Regional and district hospitals	• All district hospitals, as part of the neonatology outreach	• Varied, depending on districts targeted for newborn care training
**Existence of national KMC guidelines**	• Yes	• Incorporated in newborn care guidelines	• Yes	• Incorporated in newborn care guidelines
**Location of KMC services at the health facility**	• Services mostly provided as part of postnatal care<br>• Separate KMC room: 11/14	• Services provided as part of postnatal, paediatric or neonatal care<br>• Separate KMC room: 4/7	• Services provided as part of maternity or neonatal care<br>• Separate KMC room: 7/7	• Services mostly provided as part of postnatal care<br>• Separate KMC room: 6/11
**Availability of KMC education materials**	• Posters: 11/14; Murals: 2/14; Leaflets: 1/14; Counselling cards: 3/14; Photocopies on wall: 1/14	• None	• Photocopies on wall: 1/7	• Posters by donors: 7/11; Own posters: 2/11
**Awareness of KMC being promoted in antenatal care services**	• 6/14	• 1/7	• 0/7	• 3/11
**Promotion of intermittent KMC for babies not yet admitted for continuous KMC**	• 9/14. No facilities have a set schedule or recording system	• 2/7. No facilities have a set schedule or recording system	• 3/7. No facilities have a set schedule or recording system	• 6/11. No facilities have a set schedule, 2 facilities recorded hours per day in KMC
**Written policy for feeding low birth weight babies**	• 3/14	• 4/7	• Incorporated in national neonatal protocols<br>• Available: 7/7	• 3/11
**Monitors' impression of the quality of documentation (availability, completeness)**	• Good: 4<br>• Average: 6<br>• Poor: 4	• Good: 2<br>• Average: 4<br>• Poor: 1	• Good: 2<br>• Average: 5<br>• Poor: 0	• Good: 1<br>• Average: 6<br>• Poor: 4

**Availability of KMC register**	• 10/14	• 7/7	• 5/7	• 7/11
**Number of staff trained in KMC**	• Total: 780<br>• In facilities visited: 238 (range 0-155)	• Total: Unknown<br>• In facilities visited: 142 (range 2-71)	• Total: 501<br>• In facilities visited: 60 (range 3-14)	• Total: Unknown<br>• In facilities visited: 262 (range 1-60)
**KMC orientation for new staff**	• 3/14	• 2/7	• 6/7	• 5/11
**Evidence of follow-up system (documentation, schedule)**	• Good evidence: 3<br>• Partial evidence: 9<br>• Little/no evidence: 2	• Good evidence: 4<br>• Partial evidence: 3<br>• Little/no evidence: 0	• Good evidence: 5<br>• Partial evidence: 2<br>• Little/no evidence: 0	• Good evidence: 4<br>• Partial evidence: 2<br>• Little/no evidence: 5

Expanded table of key findings from hospitals visited

Topic	Malawi	Mali	Rwanda	Uganda
Facilities targeted for implementation	• All health facilities:<br>District hospitals and selected community health centres<br>Central and mission hospitals	• Regional and district hospitals as funding and buy-in allowed	• All district hospitals	• Eclectic, depending on districts targeted for newborn care training
National KMC guidelines	• Yes	• Incorporated in newborn care guidelines	• Yes	• Incorporated in newborn care guidelines
Number, location and types of facilities visited	• 14 visited<br>• All levels<br>• All 3 regions<br>• Breakdown:<br>1 central teaching hospital<br>9 district hospitals<br>1 mission hospital<br>1 rural hospital<br>2 community health centres	• 7 visited<br>• All levels<br>• 3 regions in south of country<br>• Breakdown:<br>• 1 central teaching hospital<br>• 3 regional hospitals<br>• 3 district hospitals	• 7 visited<br>• District level only<br>• 4/5 provinces<br>• Breakdown:<br>7 district hospitals	• 11 visited<br>• All levels<br>• 4/9 regions<br>• Breakdown:<br>1 central teaching hospital<br>1 regional hospital<br>4 district hospitals<br>3 no-for-profit hospitals<br>2 health centres IV

Topic	Malawi	Mali	Rwanda	Uganda
Baby-friendly Hospital Initiative in visited health facilities	• 9/14 facilities designated as baby-friendly<br>• Most obtained status for first time between 2004 and 2008<br>• No subsequent re-assessments	• 4/7 claimed baby-friendly status<br>• No recall of process or year of accreditation<br>• No visible onsite evidence of baby-friendly status	• 0/7<br>• One facility: preparations for assessment made	• 1/11 facilities assessed 2005<br>• 2/11 assessed end 2011 – no results at time of visit
Facilities	• General vision and mission statement displayed: 3 district hospitals<br><br>• Vision and mission statement for neonatal / maternity unit:<br>Formulated: 0 facilities<br><br>• Overnight facilities for mothers not yet doing KMC or doing intermittent KMC: 9/14<br>Floor sleeping in some facilities<br><br>• Location of KMC: Mostly part of maternity unit (linked to postnatal ward)<br><br>• Separate KMC or side room: 11/14<br>Rest: beds in postnatal ward<br><br>• Number of dedicated KMC beds	• General vision and mission statement displayed: 0 facilities<br><br>• Vision and mission statement for neonatal / maternity unit:<br>Formulated: 0 facilities<br><br>• Overnight facilities for mothers not yet doing KMC or doing intermittent KMC: 4/7<br>Accommodation often conditional to space being available<br><br>• Location of KMC: Part of postnatal, paediatric or neonatology wards<br><br>• Separate KMC room: 4/7<br>Rest: beds in postnatal ward<br><br>• Number of dedicated KMC beds	• General vision and mission statement displayed: 0 facilities<br><br>• Vision and mission statement for neonatal / maternity unit :<br>Formulated: National neonatology guidelines<br>Displayed: 0 facilities<br><br>• Overnight facilities for mothers not yet doing KMC or doing intermittent KMC: 7/7<br>Overcrowding in postnatal ward - mothers sharing beds<br><br>• Location of KMC: Part of neonatology or combined maternity-neonatology services<br><br>• Separate KMC room: 7/7<br><br>• Number of dedicated KMC beds	• General vision, mission and value statement displayed: 5 hospitals<br><br>• Vision and mission statement for neonatal / maternity unit:<br>Formulated: 0 facilities<br><br>• Overnight facilities for mothers not yet doing KMC or doing intermittent KMC: 11/11<br>Floor sleeping in some facilities<br>Use of maternity waiting house in one hospital<br><br>• Location of KMC: Mostly part of maternity unit (linked to postnatal ward)<br><br>• Separate KMC room: 6/11<br>Two with space in postnatal ward and in a corridor separated by curtains<br>Rest: beds in postnatal ward<br><br>• Number of dedicated KMC beds

Topic	Malawi	Mali	Rwanda	Uganda
	per facility:<br>1-10 (mostly 3-7)<br><br>• Continuous-KMC environment:<br>KMC space: pleasant to cramped or unattractive<br>Low beds: 7/14<br>Comfortable chairs: 4/14<br>Back rest or pillows for bed: 12/14<br>Provision of linen: very few<br>Storage space for mothers: very few<br><br>• KMC educational materials:<br>✔ Donor posters: 11<br>✔ Own posters: 4<br>✔ Murals: 2<br>✔ Leaflets: 1<br>✔ Counselling cards: 3<br>✔ Photocopies on wall: 1<br><br>• Food provided for mothers:<br>All public hospitals, but not in all mission hospitals or community health centres	per facility:<br>2-10<br><br>• Continuous-KMC environment:<br>KMC space: pleasant to cramped or unattractive<br>Low beds: 7/7<br>Comfortable chairs: 2/7<br>Back rest or pillows for bed: 5/7<br>Provision of linen: very few<br>Storage space for mothers: very few<br><br>• KMC educational materials:<br>Donor posters: 0<br>Own posters: 0<br>Murals: 0<br>Leaflets: 0<br>Counselling cards: 0<br>Photocopies on wall: 0<br><br>• Food provided for mothers:<br>No	per facility:<br>0-8 (mostly 4-5<br>In one facilities KMC provided in recliner chairs)<br><br>• Continuous-KMC environment:<br>KMC space: pleasant to cramped or unattractive<br>Surgical beds: 7/7<br>Comfortable chairs: 7/7<br>Back rest or pillows for bed: 6/6<br>Provision of linen: very few<br>Storage space for mothers: very few<br><br>• KMC educational materials:<br>Donor posters: 0<br>Own posters: 0<br>Murals: 0<br>Leaflets: 0<br>Counselling cards: 0<br>✔ Photocopies on wall: 1<br><br>• Food provided for mothers:<br>No	per facility:<br>1-6<br><br>• Continuous-KMC environment:<br>KMC space: pleasant to cramped or unattractive<br>Surgical beds: 8/8<br>Comfortable chairs: 5/11<br>Back rest or pillows for bed: 3/11<br>Provision of linen: very few<br>Storage space for mothers: very few<br><br>• KMC educational materials:<br>✔ Donor posters: 7<br>✔ Own posters: 2<br>Murals: 0<br>Leaflets: 0<br>Counselling cards: 0<br>Photocopies on wall: 0<br><br>• Food provided for mothers:<br>Not in public health facilities
Practice	• Self-report on type of KMC provided:<br>Intermittent: 9/11 hospitals<br>"Continuous": 14/14<br>Verified continuous: 11/14	• Self-report on type of KMC provided:<br>Intermittent: 2/7<br>"Continuous": 7/7<br>Verified continuous: Not established	• Self-report on type of KMC provided:<br>Intermittent: 3/7<br>"Continuous": 7/7<br>Verified continuous: Not established	• Self-report on type of KMC provided:<br>Intermittent: 6/9 hospitals<br>"Continuous": 8/11<br>Verified continuous: 3/8

Topic	Malawi	Mali	Rwanda	Uganda
	• Reasons baby taken out of (continuous) KMC:<br>✔ Feeding (breast/cup)<br>✔ Nappy change / clean baby<br>✔ Mother meals<br>✔ Mother toilet/bath<br>Mother laundry<br>Mother must go out<br>✔ No guardian around<br>✔ Mother tired<br>Mother in pain<br>✔ Reluctance of mother<br><br>• Decision readiness for KMC:<br>Clinical Officer: 0<br>Nurse(s): 14<br>Joint CO & nurse(s): 4<br><br>• Awareness of KMC being promoted in antenatal care services: 6/14<br><br>• Transport to facility in KMC position:<br>Always 3<br>Sometimes 7<br>Seldom, never 3<br>No experience 1<br><br>• Transport from facility in KMC position:<br>Always 11<br>Sometimes 2<br>Seldom, never 0<br>No experience 1	• Reasons baby taken out of (continuous) KMC:<br>✔ Feeding (breast/cup)<br>Nappy change / clean baby<br>✔ Mother meals<br>✔ Mother toilet/bath<br>Mother laundry<br>✔ Mother must go out<br>No guardian around<br>✔ Mother tired<br>✔ Mother in pain<br>✔ Reluctance of mother<br><br>• Decision readiness for KMC:<br>Doctor: 5<br>Nurse(s): 2<br>Joint doctor & nurse(s): 7<br><br>• Awareness of KMC being promoted in antenatal care services: 1/7<br><br>• Transport to facility in KMC position:<br>Always 0<br>Sometimes 0<br>Seldom, never 1<br>No experience 6<br><br>• Transport from facility in KMC position:<br>Always 2<br>Sometimes 1<br>Seldom, never 2<br>No experience 2	• Reasons baby taken out of (continuous) KMC:<br>Feeding (breast/cup)<br>Nappy change / clean baby<br>✔ Mother meals<br>✔ Mother toilet/bath<br>✔ Mother laundry<br>Mother must go out<br>✔ No guardian around<br>✔ Mother tired<br>Mother in pain<br>✔ Reluctance of mother<br><br>• Decision readiness for KMC:<br>Doctor: 6<br>Nurse(s): 1<br>Joint doctor & nurse(s): 2<br><br>• Awareness of KMC being promoted in antenatal care services: 0/7<br><br>• Transport to facility in KMC position:<br>Always 1<br>Sometimes 4<br>Seldom, never 1<br>No experience 1<br><br>• Transport from facility in KMC position:<br>Always 2<br>Sometimes 3<br>Seldom, never 1<br>No experience 1	• Reasons baby taken out of (continuous) KMC:<br>✔ Feeding (breast/cup)<br>✔ Nappy change / clean baby<br>✔ Mother meals<br>✔ Mother toilet/bath<br>✔ Mother laundry<br>✔ Mother must go out<br>No guardian around<br>✔ Mother tired<br>✔ Mother in pain<br>Reluctance of mother<br><br>• Decision readiness for KMC:<br>Doctor: 8<br>Nurse(s): 3<br>Joint doctor & nurse(s): 7<br><br>• Awareness of KMC being promoted in antenatal care services: 3/11<br><br>• Transport to facility in KMC position:<br>Always 0<br>Sometimes 2<br>Seldom, never 9<br>No experience 0<br><br>• Transport from facility in KMC position:<br>Always 3<br>Sometimes 3<br>Seldom, never 5<br>No experience 0

Topic	Malawi	Mali	Rwanda	Uganda
Position	• Babies observed in KMC position: 8/14<br>• Recording of intermittent KMC: 0/9<br>• Schedule / Programme for intermittent KMC: 0/9<br>• Mothers' diligence of KMC practice<br>Diligent: 7/14<br>Some KMC: 3/14<br>Little KMC: 2/14<br>Not able to judge: 2/14<br>• Tying methods:<br>✔ Local cloth<br>Lycra band<br>✔ Special blouse<br>Square with bands<br>Kalafong thari<br>Sheets<br>• Movement of mother with baby in KMC:<br>✔ Only in KMC room (2)<br>✔ In neonatal unit/maternity<br>✔ Within hospital premises (2)	• Babies observed in KMC position: 2/7<br>• Recording of intermittent KMC: 0/2<br>• Schedule / Programme for intermittent KMC: 0/2<br>• Mothers' diligence of KMC practice<br>Diligent: 1/7<br>Some KMC: 0/7<br>Little KMC: 0/7<br>Not able to judge: 6/7<br>• Tying methods:<br>✔ Local cloth<br>✔ Lycra band<br>Special blouse<br>✔ Square with bands<br>Kalafong thari<br>Sheets<br>• Movement of mother with baby in KMC:<br>Only in KMC room<br>✔ In paediatric services<br>✔ Within hospital premises (2)	• Babies observed in KMC position: 7/7<br>• Recording of intermittent KMC: 0/3<br>• Schedule / Programme for intermittent KMC: 0/3<br>• Mothers' diligence of KMC practice<br>Diligent: 6/7<br>Some KMC: 0/7<br>Little KMC: 1/7<br>Not able to judge: 0/7<br>• Tying methods:<br>✔ Local cloth<br>Lycra band<br>Special blouse<br>Square with bands<br>✔ Kalafong thari<br>Sheets<br>• Movement of mother with baby in KMC:<br>Only in KMC room<br>✔ In neonatal unit/maternity<br>✔ Within hospital premises	• Babies observed in KMC position: 6/11<br>• Recording of intermittent KMC: 2/6<br>• Schedule / Programme for intermittent KMC: 0/6<br>• Mothers' diligence of KMC practice<br>Diligent: 3/11<br>Some KMC: 4/11<br>Little KMC: 1/11<br>Not able to judge: 3/11<br>• Tying methods:<br>✔ Local cloth<br>Lycra band<br>Special blouse<br>Square with bands<br>✔ Kalafong thari<br>✔ Sheets<br>• Movement of mother with baby in KMC:<br>✔ Only in KMC room<br>✔ In neonatal unit/maternity<br>Within hospital premises

Topic	Malawi	Mali	Rwanda	Uganda
	• Role of guardian / companion for mother:<br>Guardian allowed: 8/14<br>Unrestricted access: 3/8<br>Tasks:<br>✔ Psychological support<br>Helping mother with position<br>✔ Carrying baby in KMC<br>✔ Twins<br>✔ Caesarean section<br>✔ Illness<br>✔ Spoon of cup feeding<br>✔ Changing nappies<br>✔ Collecting medication<br>✔ Meals / Bringing food<br>Washing mother's clothes<br>✔ Motivator of KMC to mother<br>✔ Father as support person	• Role of guardian / companion for mother:<br>Guardian allowed: 7/7<br>Unrestricted access: 3/8<br>Tasks:<br>✔ Psychological support<br>✔ Helping mother with position<br>✔ Carrying baby in KMC<br>Twins<br>Caesarean section<br>Illness<br>✔ Spoon of cup feeding<br>Changing nappies<br>✔ Collecting medication<br>✔ Meals / Bringing food<br>✔ Washing mother's clothes<br>✔ Motivator of KMC to mother<br>Father as support person	• Role of guardian / companion for mother:<br>• Guardian allowed: 6/7<br>Unrestricted access: 2/7<br>Tasks:<br>Psychological support<br>Helping mother with position<br>✔ Carrying baby in KMC<br>✔ Twins<br>Caesarean section<br>✔ Illness (skin infection)<br>Spoon of cup feeding<br>Changing nappies<br>Collecting medication<br>✔ Meals / Bringing food<br>✔ Washing mother's clothes<br>Motivator of KMC to mother<br>✔ Father as support person	• Role of guardian / companion for mother:<br>• Guardian allowed: 8/11<br>Unrestricted access: 3/8<br>Tasks:<br>✔ Psychological support<br>Helping mother with position<br>Carrying baby in KMC<br>✔ Twins<br>✔ Caesarean section<br>Illness<br>Spoon of cup feeding<br>Changing nappies<br>Collecting medication<br>✔ Meals / Bringing food<br>✔Washing mother's clothes<br>Motivator of KMC to mother<br>✔ Father as support person
Nutrition (feeding and weight monitoring)	• Written feeding policy: 3/14<br>• Copy of policy provided: 2/3<br>• Feeding job aid for calculating volumes of feeds:<br>11/11 hospitals<br>• Records of each EBM feed: 3/14	• Written feeding policy: 4/7<br>• Copy of policy provided: 4/4<br>• Feeding job aid for calculating volumes of feeds:<br>5/7 hospitals<br>• Records of each EBM feed: 3/7	• Written feeding policy: Incorporated in national neonatal protocols<br>• Copy of policy provided: Printed document<br>• Feeding job aid for calculating volumes of feeds:<br>Part of neonatal protocols (displayed in 3 hospitals)<br>• Records of each EBM feed: 4/7	• Written feeding policy: 3/11<br>• Copy of policy provided: 3/3<br>• Feeding job aid for calculating volumes of feeds:<br>6/9 hospitals<br>• Records of each EBM feed: 3/9

Topic	Malawi	Mali	Rwanda	Uganda
	• Regular weighing of babies: 11/14<br>Once per day: 0<br>Twice per week: 1<br><br>• Recording of weight:<br>✔ Baby's observation sheet<br>✔ Baby's file (nursing/doctors notes)<br>✔ Feeding chart<br>✔ KMC register<br>Daily weight register<br>✔ Labour chart<br>Baby's health passport<br>✔ Mother's health passport	• Regular weighing of babies: 7/7<br>Once per day: 7<br><br>• Recording of weight:<br>✔ Baby's observation sheet<br>Baby's file (nursing/doctors notes)<br>Feeding chart<br>✔ KMC register<br>Daily weight register<br>Labour chart<br>✔ Baby's health passport<br>Mother's health passport	• Regular weighing of babies: 7/7<br>Once per day: 7<br><br>• Recording of weight:<br>✔ Baby's observation sheet<br>Baby's file (nursing/doctors notes)<br>Feeding chart<br>KMC register<br>✔ Daily weight register<br>Labour chart<br>Baby's health passport<br>Mother's health passport	• Regular weighing of babies: 7/11<br>Once per day: 10<br>Once every 2 days: 2<br>Once per week: 1<br><br>• Recording of weight:<br>✔ Baby's observation sheet<br>✔ Baby's file (nursing/doctors notes)<br>Feeding chart<br>KMC register<br>Daily weight register<br>✔ Mother's chart<br>Baby's health passport<br>Mother's health passport
Documentation and record keeping (availability, completeness)	• Quality of data:<br>Good: 4<br>Average: 6<br>Poor: 4<br><br>• Special collective KMC register (printed or handwritten): 10/14<br><br>• Other records with KMC evidence:<br>✔ Baby's observation sheet (3)<br>✔ Nurses' or doctors' notes (4)<br>✔ Baby's health passport (10)<br>Discharge letter	• Quality of data:<br>Good: 2<br>Average: 4<br>Poor: 1<br><br>• Special collective KMC register (printed or handwritten): 7/7<br><br>• Other records with KMC evidence:<br>✔ Baby's observation sheet (5)<br>✔ Nurses' or doctors' notes<br>✔ Baby's health passport (6)<br>Discharge letter	• Quality of data:<br>Good: 2<br>Average: 5<br>Poor: 0<br><br>• Special collective KMC register (printed or handwritten): 5/7<br><br>• Other records with KMC evidence:<br>✔ Baby's observation sheet (6)<br>✔ Nurses' or doctors' notes (4)<br>Baby's health passport<br>Discharge letter	• Quality of data:<br>Good: 1<br>Average: 6<br>Poor: 4<br><br>• Special collective KMC register (printed or handwritten): 7/11<br><br>• Other records with KMC evidence:<br>Baby's observation sheet<br>✔ Nurses' or doctors' notes (4)<br>✔ Baby's health passport (1)<br>✔ Discharge letter (1)

Topic	Malawi	Mali	Rwanda	Uganda
	• Summary figures for at least one year: 13/14<br>No breakdown continuous and intermittent KMC<br>LBW babies with and without KMC grouped together<br>Poor recording – accuracy of audit?<br><br>• Written documents to contribute to sustainability:<br>Checklist procedures on admission (0)<br>✔ Guidelines for practice of KMC / LBW (9)<br>✔ Criteria for admission & re-admission (4)<br>✔ Discharge criteria (2)<br>✔ Daily monitoring (1)<br>✔ Daily counselling (1)	• Summary figures for at least one year: 3/7<br>Poor recording – accuracy of audit?<br><br>• Written documents to contribute to sustainability:<br>Checklist procedures on admission (0)<br>✔ Guidelines for practice of KMC / LBW (2)<br>Criteria for admission & re-admission<br>Discharge criteria<br>Daily monitoring<br>Daily counselling	• Summary figures for at least one year: 4/7<br>No breakdown continuous and intermittent KMC<br>LBW babies with and without KMC grouped together<br><br>• Written documents to contribute to sustainability:<br>Checklist procedures on admission (0)<br>✔ Guidelines for practice of KMC / LBW (7)*<br>✔ Criteria for admission & re-admission (7)*<br>✔ Discharge criteria (7)*<br>✔ Daily monitoring (7)*<br>✔ Daily counselling<br>* Included in national neonatal protocols	• Summary figures for at least one year: 4/7<br><br>• Written documents to contribute to sustainability:<br>✔ Checklist procedures on admission (2)<br>✔ Guidelines for practice of KMC / LBW (2)<br>Criteria for admission & re-admission<br>Discharge criteria<br>Daily monitoring<br>Daily counselling
Staff	• Total of known number of health workers trained in KMC: 780<br>A lot of training integrated in ENC, IMNC and EMOnC<br><br>• Total of known number trained in facilities visited: 238<br>Range per facility: 0-155	• Total of known number of health workers trained in KMC: Unknown<br><br>• Total of known number trained in facilities visited: 142<br>Range per facility: 2-71<br>Problem of staff leaving shortly after having been trained – loss of continuity and skills	• Total of known number of health workers trained in KMC: 501<br>A lot of training integrated in ENC, IMNC and EMOnC<br><br>• Total of known number trained in facilities visited: 60<br>Range per facility: 3-14	• Total of known number of health workers trained in KMC: Unknown<br>A lot of training integrated in ENC, IMNC and EMOnC<br><br>• Total of known number trained in facilities visited: 262 (under-estimate)<br>Range per facility:1-60

Topic	Malawi	Mali	Rwanda	Uganda
	• Long-term plan for KMC training: 8/14<br>• Plan in writing: 3/8<br><br>• Staff allocations:<br>Rotation of nurses: 10/14<br>No night duty for registered nurses: 2/10<br>KMC wards not adequately supervised, especially at night<br><br>• Special KMC orientation programme for new staff: 3/14<br><br>• Practical training for students: 12/14<br>Nursing: 11/12<br>Medical & related: 8/12<br>• Not all (especially junior) students rotate through KMC	• Long-term plan for KMC training: 2/7<br>• Plan in writing: 1/2<br><br>• Staff allocations:<br>Rotation of nurses: 0/7<br><br>• Special KMC orientation programme for new staff: 2/7<br><br>• Practical training for students: 7/7<br>Nursing: 7/7<br>Medical: 6/7	• Long-term plan for KMC training: 6/7<br>• Plan in writing: 0/6<br><br>• Staff allocations:<br>Rotation of nurses: 1/7<br><br>• Special KMC orientation programme for new staff: 6/7<br><br>• Practical training for students: 5/7<br>Nursing: 5/5<br>Medical: 5/5	• Long-term plan for KMC training: 3/11<br>• Plan in writing: 1/3<br><br>• Staff allocations:<br>Rotation of nurses: 11/11<br><br>• Special KMC orientation programme for new staff: 5/11<br><br>• Practical training for students: 9/11<br>Nursing: 8/9<br>Medical: 5/9<br>• Not all (especially junior) students rotate through KMC
Discharge and follow-up	• Decision readiness for discharge:<br>Clinical Officer: 0<br>Nurse(s): 14<br>Joint CO & nurse(s): 5<br>Mother's request: 0<br><br>• Evidence of follow-up system (documentation, schedule, etc):<br>Good evidence: 3<br>Partial evidence: 9<br>Little / No evidence: 2	• Decision readiness for discharge:<br>Doctor: 6<br>Nurse(s): 1<br>Joint doctor & nurse(s): 3<br>Mother's request: 0<br><br>• Evidence of follow-up system (documentation, schedule, etc):<br>Good evidence: 4<br>Partial evidence: 3<br>Little / No evidence: 0	• Decision readiness for discharge:<br>Doctor: 7<br>Nurse(s): 0<br>Joint doctor & nurse(s): 1<br>Mother's request: 0<br><br>• Evidence of follow-up system (documentation, schedule, etc):<br>Good evidence: 5<br>Partial evidence: 2<br>Little / No evidence: 0	• Decision readiness for discharge:<br>Doctor: 8<br>Nurse(s): 3<br>Joint doctor & nurse(s): 7<br>Mother's request: 5<br><br>• Evidence of follow-up system (documentation, schedule, etc):<br>Good evidence: 4<br>Partial evidence: 2<br>Little / No evidence: 5

Topic	Malawi	Mali	Rwanda	Uganda
	• Location of initial follow-up: Mostly at facility where born or receiving KMC*<br>In KMC space (7)<br>In neonatal unit (1)<br>Paediatric outpatients Maternity ward (4)<br>* Excluding 2 community health centres	• Location of initial follow-up: Mostly at facility where born or receiving KMC<br>✔ In KMC space (4)<br>✔ In neonatal unit (2)<br>✔ Paediatric outpatients (1)<br>Maternity ward	• Location of initial follow-up: Mostly at facility where born or receiving KMC<br>✔ In KMC space (2)<br>✔ In neonatal unit (3)<br>✔ Paediatric outpatients (2)<br>Maternity ward	• Location of initial follow-up: Mostly at facility where born or receiving KMC<br>In KMC space<br>✔ In neonatal unit (4)<br>✔ Paediatric outpatients (3)<br>✔ Maternity ward (4)
	• Records of follow-up visits: 12/14<br>✔ Register (date & weight only) (10)<br>✔ Baby's health passport (1)<br>✔ Special follow-up form (1)<br>Baby's original file<br>Doctor's notes	• Records of follow-up visits: 6/7<br>Register (date & weight only)<br>Baby's health passport<br>✔ Special follow-up form (6)<br>Baby's original file<br>Doctor's notes	• Records of follow-up visits: 5/7<br>✔ Register (date & weight only) (3)<br>Baby's health passport<br>Special follow-up form (1)<br>✔ Baby's original file (1)<br>Doctor's notes	• Records of follow-up visits: 6/11<br>✔ Register (date & weight only) (3)<br>Baby's health passport<br>Special follow-up form<br>✔ Baby's original file (1)<br>✔ Doctor's notes (2)<br>Mostly no distinction between LBW and KMC babies in records
	• End-point of special review:<br>Weight: mostly 2500 g<br>Age: -<br>Number of visits: -	• End-point of special review:<br>Weight: 2500 g (2)<br>Age:<br>1 year corrected age (3)<br>18 months (1)<br>2 years (1)<br>Number of visits: -	• End-point of special review:<br>Weight: 2000 g – 2500 g (4)<br>Age: -<br>Number of visits:<br>1 visits (1)<br>4 visits (2)	• End-point of special review:<br>Weight: 2000 – 3000 g (Mostly 2500 g)<br>Age:<br>2 years (1)<br>Number of visits:<br>3 visits (1)
	• Estimates of follow-up rates: range of 50-100% (one = 5%)	• Estimates of follow-up rates: range of 20-98%	• Estimates of follow-up rates: range of <50-100%	• Estimates of follow-up rates: range of 30-95%

Topic	Malawi	Mali	Rwanda	Uganda
	• Home visits:<br>5 hospitals<br>HSAs linked to health centres<br>Mostly for tracing defaulters	• Home visits:<br>None	• Home visits:<br>None	• Home visits:<br>1 hospital (research study)<br>3 informal visits<br>Mostly for tracing defaulters
	• Methods of communication with health centres:<br>None<br>Phone<br>✔ Referral/discharge slip (6)<br>✔ Baby's health passport (5)<br>✔ Mother provided with contact number of HSAs (1)	• Methods of communication with health centres:<br>✔ None (3)<br>Phone<br>✔ Referral/discharge slip (4)<br>Baby's health passport<br>Mother provided with contact number of nearest community health worker	• Methods of communication with health centres:<br>✔ None (4)<br>✔ Phone (1)<br>✔ Referral/discharge slip (2)<br>Baby's health passport<br>Mother provided with contact number of ASM	• Methods of communication with health centres:<br>✔ None (5)<br>✔ Phone (2)<br>✔ Referral/discharge slip (4)<br>Baby's health passport<br>Mother provided with contact number of nearest community health worker

Topic	Malawi	Mali	Rwanda	Uganda
	• Measures to encourage follow-up:<br>None<br>✔ Providing follow-up dates<br>✔ Importance of follow-up (e.g. vaccinations, growth monitoring)<br>✔ Notes in baby's health passport<br>✔ Good relationship of staff with mothers<br>✔ Praise for good care<br>✔ Threat tactics (1)<br>✔ Incentives (e.g. baby hats)<br>✔ Free hospital stay and follow-up<br>Phone calls<br>✔ Referral to HSA	• Measures to encourage follow-up:<br>None<br>Providing follow-up dates<br>✔ Importance of follow-up (e.g. vaccinations, growth monitoring) (6)<br>Notes in baby's health passport<br>Good relationship of staff with mothers<br>Praise for good care<br>Threat tactics<br>✔ Incentives (e.g. KMC pouch)<br>✔ Free hospital stay and follow-up<br>✔ Phone calls (to defaulters)<br>Referral to community health workers	• Measures to encourage follow-up:<br>None<br>Providing follow-up dates<br>✔ Importance of follow-up (e.g. vaccinations, growth monitoring)<br>Notes in baby's health passport<br>Good relationship of staff with mothers<br>Praise for good care<br>Threat tactics<br>Incentives<br>Free hospital stay and follow-up<br>Phone calls<br>✔ Referral to ASM (2)	• Measures to encourage follow-up:<br>✔ None<br>✔ Providing follow-up dates<br>Importance of follow-up (e.g. vaccinations, growth monitoring)<br>Notes in baby's health passport<br>Good relationship of staff with mothers<br>Praise for good care<br>Threat tactics<br>Incentives (e.g. baby hats)<br>Free hospital stay and follow-up<br>✔ Phone calls (from mothers)<br>Referral to community health workers<br>Know they will see hospital
Client satisfaction (not focus of evaluation)	• Not probed	• Not probed	• Not probed	• Not probed<br>• One private-not-for-profit hospital had a book where clients could write comments on their KMC experience

Topic	Malawi	Mali	Rwanda	Uganda
Community involvement (not focus of evaluation)	• Training of health surveillance assistants (HSAs) in newborn care<br>• In at least one of the learning districts	• No specific training action directed from the national level	• Initiation of *agents de santé maternelle* (ASMs)<br>• KMC part of ASM package	• Eclectic, depending on which districts were targeted for training in newborn care<br>• Uganda Newborn Study (UNEST) in one district linked implementation with research that included community health workers
	• Great variations among districts<br>• Grandmothers as promoters of KMC in at least one district<br>• Advocacy activities and sensitisation of traditional leaders in some districts	• Some training and sensitisation activities in one district	• Not probed in depth	• Unknown
